# A silicon metal-oxide-semiconductor electron spin-orbit qubit

**DOI:** 10.1038/s41467-018-04200-0

**Published:** 2018-05-02

**Authors:** Ryan M. Jock, N. Tobias Jacobson, Patrick Harvey-Collard, Andrew M. Mounce, Vanita Srinivasa, Dan R. Ward, John Anderson, Ron Manginell, Joel R. Wendt, Martin Rudolph, Tammy Pluym, John King Gamble, Andrew D. Baczewski, Wayne M. Witzel, Malcolm S. Carroll

**Affiliations:** 1 Sandia National Laboratories, Albuquerque, NM 87185 USA; 2 Center for Computing Research, Sandia National Laboratories, Albuquerque, NM 87185 USA; 3 Département de Physique et Institut Quantique, Université de Sherbrooke, 2500 boul. de l’Université, Sherbrooke, QC J1K 2R1 Canada

## Abstract

The silicon metal-oxide-semiconductor (MOS) material system is a technologically important implementation of spin-based quantum information processing. However, the MOS interface is imperfect leading to concerns about 1/*f* trap noise and variability in the electron *g*-factor due to spin–orbit (SO) effects. Here we advantageously use interface–SO coupling for a critical control axis in a double-quantum-dot singlet–triplet qubit. The magnetic field-orientation dependence of the *g*-factors is consistent with Rashba and Dresselhaus interface–SO contributions. The resulting all-electrical, two-axis control is also used to probe the MOS interface noise. The measured inhomogeneous dephasing time, $T_{{\mathrm{2m}}}^ \star$, of 1.6 μs is consistent with 99.95% ^28^Si enrichment. Furthermore, when tuned to be sensitive to exchange fluctuations, a quasi-static charge noise detuning variance of 2 μeV is observed, competitive with low-noise reports in other semiconductor qubits. This work, therefore, demonstrates that the MOS interface inherently provides properties for two-axis qubit control, while not increasing noise relative to other material choices.

## Abstract (web summary)

As the performance of silicon-based qubits has improved, there has been increasing focus on developing designs that are compatible with industrial processes. Here, Jock et al. exploit spin-orbit coupling to demonstrate full, all-electrical control of a metal-oxide-semiconductor electron spin qubit.

## Introduction

Spin qubits in silicon metal-oxide-semiconductor (MOS) structures offer a promising path towards implementing quantum information processing. The MOS system combined with enriched ^28^Si provides a magnetic vacuum^1^ and promises to leverage the extensive complementary metal-oxide-semiconductor (CMOS) fabrication platform. Recently, several critical demonstrations have shown long spin coherence times^2^, two-qubit couplings of single spins in a multi-quantum dot layout^3^, large tunable valley-splitting^2,4,5^, and importantly similar valley splittings in different process flows and multiple devices^4,5^. Yet, there are persistent concerns about the intrinsically imperfect Si/SiO_2_ interface produces persistent concerns about charge noise from the disordered interface. Two potentially key performance challenges identified are extra detrimental charge noise and variable *g*-factors^6,7^.

Charge traps and two-level fluctuators near the interface are believed to be potential sources of noise in MOS devices^8–10^. To attempt to suppress the challenges of disorder and trap noise Si quantum dot (QD) spin qubits have also been developed in heteroepitaxial Si/SiGe^11–16^. The imperfect crystal–dielectric interface is shifted further away. This is the predominant choice despite reports of difficulties with small or variable valley-splitting^13–15,17^. Nevertheless qubits have successfully been demonstrated and charge noise has been studied in Si/SiGe qubits^12,16,18,19^, but only indirect measures of charge noise in MOS qubits have been reported^3,20,21^. Direct characterization of charge noise at the MOS interface is needed for comparison.

**Fig. 1:**
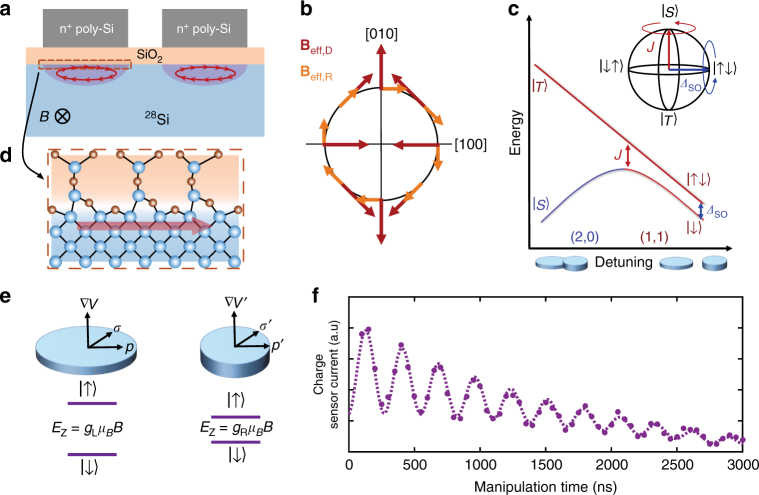
MOS spin–orbit-driven singlet–triplet qubit. **a** Cartoon representation of the interface spin–orbit interaction. For an electron confined to a QD, an in-plane magnetic field will cause a finite momentum at the interface which, in the presence of broken inversion symmetry, leads to a spin–orbit interaction. The position of the QDs presented in this work, relative to the gates, differs from what is portrayed here (see Supplementary Fig. 2). **b** Schematic example of the effective spin–orbit field due to the Dresselhaus (red) and Rashba (orange) interactions for in-plane electron momentum. **c** Schematic energy diagram of the DQD near the (2, 0) → (1, 1) charge transition, showing the energy of the singlet and triplet states as a function of QD–QD detuning, $\epsilon$. Near the interdot transition ($\epsilon$ = 0), the exchange energy, *J*, dominates the electronic interaction and drives rotations about the *Z*-axis (red arrow in inset). Deep into the (1, 1) charge sector ($\epsilon$ > 0), *J* is small and the electronic states rotate about the *X*-axis due to a difference in Zeeman energy between each QD (blue arrow in inset). **d** Details of the interface at the inter-atomic bond level govern the spin–orbit interaction. **e** The local electrostatic environment of each QD leads to different momenta and electric fields at the interface and, thus, distinct spin–orbit interactions and Zeeman energy splitting. **f** Charge sensor current as a function of time spent deep in the (1, 1) charge sector, where higher current indicates a higher probability of measuring a singlet state. The oscillations indicate clear *X*-rotations due to a difference in spin–orbit interaction in each QD

Variability in *g*-factors recently observed in silicon QDs is also feared to introduce potentially challenging complications for many qubit device architectures^7^. In bulk Si, the spin–orbit (SO) interaction leads to only weakly perturbed electron *g*-factors that are close to *g* = 2. However, the inversion asymmetry of the crystal at an interface leads to a SO interaction^22–25^, as shown in Fig. 1. When a magnetic field is applied with a component parallel to the interface, electron cyclotron motion establishes a non-zero net momentum component along the interface (Fig. 1a). The coupling of the electron momentum perpendicular to the effective electric field at the interface produces the SO interaction. The vertical electric potential at the interface leads to a Rashba SO contribution due to structural inversion asymmetry (SIA). A second interaction, the Dresselhaus contribution, is attributed to microscopic interface inversion asymmetry (IIA)^26^, due to the largely unknown and possibly position dependent inter-atomic electric fields at the Si/SiO_2_ boundary. Recent work has attributed the variability in electron *g*-factor at silicon interfaces to SO coupling and interface disorder^2,6,27–30^. However, while the effects of vertical electric field and in-plane magnetic field direction have been observed, the full dependence on magnetic field strength and orientation has not, to date, been characterized in the MOS material system. We further note that this interface effect is not theoretically unique to Si MOS or SiGe/Si interfaces^22,23,31^ and variability in *g*-factor has also been observed in GaAs/AlGaAs QDs^32,33^, as well as holes in silicon QDs^34^. Because of its strength and angular dependence, which is similar to bulk SO effects, it is possible that the contribution of the interface effect, particularly on the Dresselhaus coupling, is under-appreciated in other systems that leverage strong SO coupling. Improved understanding of this effect has the potential to influence areas such as spintronics and the pursuit of forming topological states of matter^35,36^.

In this work, we advantageously use the inherent g-factor difference from the SO coupling at the Si/SiO_2_ interface to create a second axis of control for a double-quantum dot (DQD) singlet–triplet (ST) qubit. This first demonstration of an all-electrical, two-axis controlled qubit in MOS is used to study qubit noise and SO interaction at the dielectric interface. One of the central results of this paper is a quantitative characterization of charge noise in a MOS qubit (e.g., quasi-static detuning variance and Hahn-echo time). The magnitudes are comparable if not better than those reported for other semiconductor qubit materials like GaAs/AlGaAs and Si/SiGe. The second central result of this paper is that we demonstrate a SO ST qubit and use its coherent qubit rotations to characterize the SO interaction at the MOS interface over its full magnetic field angular dependence. We observe that, by choice of external magnetic field orientation, the intrinsic SO interaction may be maximized to drive spin rotations or canceled out, which may be important for applications where uniform spin splitting between many QDs is necessary. In particular, an out-of-plane magnetic field orientation, measured in this work, should uniformly suppress the SO effect. We additionally extend the theoretical framework for the interface Rashba–Dresselhaus coupling providing a gauge independent phenomenological effective mass description of the full angular dependence that is in quantitative agreement with experiment. This work, therefore, further advances our understanding of the silicon MOS interface as a potential state-of-the-art platform for quantum information technologies.

## Results

### SO ST qubit

The qubit in this work is formed within a MOS double-quantum dot (DQD). Two electrons are electrostatically confined within a double well potential, where the dominant interaction between the electrons can be electrically tuned between two regimes for two-axis control (Fig. 1c). When the electronic wave functions of the QDs overlap significantly, the exchange energy, *J*, dominates. When the two electrons are well separated, *J* is small and distinct Zeeman energies result from the differences in their interface–SO coupling. The difference in SO coupling leads to a variation in effective electron *g*-factors (Fig. 1e) This amounts to an effective magnetic field gradient between the QDs that can be tuned with control of the applied electric and magnetic fields. Thus, we achieve all-electrical two-axis control using native features of the MOS DQD system, avoiding the substantial fabrication complications to add a second axis of control for other Si qubit schemes.

**Fig. 2:**
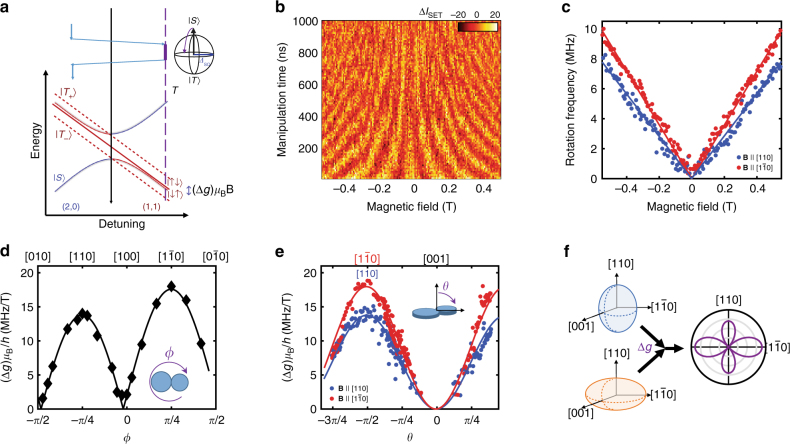
MOS interface spin–orbit interaction. **a** Energy diagram and gate pulse schematic for controlling spin–orbit rotations. We initialize the qubit into the *S*(2, 0) ground state and transfer the system to the (1, 1) charge sector with a rapid adiabatic pulse, such that it remains a singlet. The difference in Zeeman splitting between the QDs drives *X*-rotations between the *S*(1, 1) and *T*_0_(1, 1) states. A rapid adiabatic return pulse projects the states onto the *S*(2, 0) and *T*_0_(1, 1) basis for measurement. **b** Change in charge sensor current as a function of *X*-rotation manipulation time as the magnetic field is varied along the $[1\bar 10]$ crystallographic direction. **c** The extracted rotation frequency as a function of magnetic field strength along the [110] and $[1\bar 10]$ crystallographic directions. **d**,** e** Magnetic field angular dependence of the SO-driven difference in *g*-factor between the dots for the in-plane, *θ*, and out-of-plane, *ϕ*, directions, respectively. Fits to the form $({\mathrm{\Delta }}g)\mu _{\rm B}B{\mathrm{/}}h$ = $\left| {\bf{B}} \right|\left| {{\mathrm{\Delta }}\alpha - {\mathrm{\Delta }}\beta {\kern 1pt} {\mathrm{sin}}(2\phi )} \right|$ sin^2^(*θ*) are also plotted for *θ* = *π*/2 (black), *ϕ* = 3*π*/4 (blue) and *ϕ* = *π*/4 (red). **f** A cartoon representation of the angular dependence of the two QDs (left). The difference between the QD *g*-factors give an in-plane dependence represented by the cloverleaf plot on the right

We define the computational basis as the eigenstates of the two-spin system in the limit of a large singlet–triplet exchange energy, *J*. Specifically, these are the two states, *S* and *T*_0_, of the *m* = 0 subspace, which form a decoherence-free subspace relative to fluctuations in a uniform magnetic field^37^. An applied magnetic field splits the *m* = ±1 spin triplet states (*T*_±_ (1, 1)) and *m* = 0 states by the Zeeman energy *E*_Z_ = *gμ*_B_*B* to isolate the *m* = 0 subspace. A qubit state can then be initialized in a singlet ground state when the two QDs are electrically detuned out of resonance such that it is preferable to have a $( {N_{{\mathrm{QD}}_{\mathrm{1}}},N_{{\mathrm{QD}}_{\mathrm{2}}}} )$ = (2, 0) charge state (Fig. 1c). Rapid adiabatic passage to the (1, 1) charge state produces a superposition of the *S* and *T*_0_ eigenstates in the gradient field. A difference in the Larmor spin precession frequency of the two QDs induces *X*-rotations between the *S*(1, 1) and *T*_0_(1, 1) states (Figs. 1f and 2a). For each QD the angular precession frequency is given by *ω* = *gμ*_B_*B*/*ħ*, where *g* is the electron *g*-factor, *μ*_B_ is the Bohr magneton, *ħ* is Planck’s constant, and *B* is the applied magnetic field. The two-electron spin qubit will oscillate between the *S* and *T*_0_ states at a frequency 2*πf* = Δ*ω* = Δ*gμ*_B_*B*/*ħ*, where Δ*g* is the difference in electron *g*-factor between the two QDs. *Z*-rotations can be turned on by shifting the detuning closer to the charge anti-crossing where *J* is larger, driving oscillations around the equator of the Bloch sphere (Fig. 1c). The spin state is detected using Pauli blockade, combined with a remote charge sensor that detects whether the qubit state passed through the (2, 0) charge state or was blockaded in (1, 1) during the readout stage^38^.

### SO-driven spin rotations

The spin splitting of an electron in a QD is governed by an effective Zeeman Hamiltonian of the form $H_{{\mathrm{eff}}} = \frac{{\mu _{\rm B}}}{2}{\bf{B}} \cdot {\bf{g}} \cdot {\bf{\sigma }}$, where **B** is the magnetic field vector, **σ** is the vector of Pauli spin matrices (*σ*_*x*_, *σ*_*y*_, *σ*_*z*_) and **g** is the electron *g*-tensor. An electron confined to an interface will have Rashba and Dresselhaus SO couplings of the form *H*_R_ ∝ *γ*_R_(*P*_*y*_*σ*_*x*_ − *P*_*x*_*σ*_*y*_) and *H*_D_ ∝ *γ*_D_(*P*_*x*_*σ*_*x*_ − *P*_*y*_*σ*_*y*_), respectively, where *γ*_R_ and *γ*_D_ are the relative coupling strengths. The operators *σ*_*x*_, *σ*_*y*_ are Pauli spin matrices, while *P*_*x*_, *P*_*y*_ are components of the kinetic momentum **P** = −*iħ*∇ + *e***A**(**r**) along the [100], [010] direction, with *e* > 0 the elementary unit of charge and **A**(**r**) the vector potential. Including the *H*_R_ and *H*_D_ SO Hamiltonians perturbatively leads to an effective *g*-tensor of the form

1 $${\bf{g}} = \left( {\begin{array}{*{20}{c}} {g_ \bot - \frac{2}{{\mu _{\rm B}}}\alpha } & {\frac{2}{{\mu _{\rm B}}}\beta } & 0 \\ {\frac{2}{{\mu _{\rm B}}}\beta } & {g_ \bot - \frac{2}{{\mu _{\rm B}}}\alpha } & 0 \\ 0 & 0 & {g_{{\mathrm{||}}}} \end{array}} \right),$$

where *g*_⊥_ (*g*_||_) is the *g*-tensor component for the directions perpendicular (parallel) to the [001] valley of bulk silicon. Corrections to the *g*-tensor due to Rashba and Dresselhaus SO coupling are characterized by *α* and *β*, respectively. The strength of the SO interaction is predicted to depend on applied electric field, lateral confinement, valley-orbit configuration, and the atomic-scale structure of the interface (see Supplementary Note 1 and refs. ^6,27,28,30^). Consequently, the local interfacial and electrostatic environments particular to each QD produce differences in effective *g*-tensor (Fig. 2f). This will act as a difference in effective in-plane magnetic field, modifying the electron spin splitting between dots and drive rotations at a frequency

2 $$\begin{array}{*{20}{l}} {f_{{\mathrm{rot}}}(\theta ,\phi )} \hfill & = \hfill & {{\mathit{\Delta}}_{{\mathrm{SO}}}(\theta ,\phi ){\mathrm{/}}h} \hfill \\ {} \hfill & = \hfill & {({\mathrm{\Delta }}g(\theta ,\phi ))\mu _{\rm B}B{\mathrm{/}}h} \hfill \\ {} \hfill & = \hfill & {\frac{2}{h}\left| {\left\langle S \right|H\left| {T_0} \right\rangle } \right|} \hfill \\ {} \hfill & = \hfill & {\left| {\bf{B}} \right|\left| {{\mathrm{\Delta }}\alpha - {\mathrm{\Delta }}\beta {\kern 1pt} {\mathrm{sin}}(2\phi )} \right|{\mathrm{sin}}^2(\theta ),} \hfill \end{array}$$

where *ϕ* is the field direction in-plane of the interface with respect to the [100] crystallographic direction, *θ* is the out-of-plane angle relative to [001], and Δ*α* and Δ*β* quantify the difference in Rashba and Dresselhaus *g*-tensor perturbations between the two QDs, respectively. Our theoretical model for the SO coupling associated with the interface is discussed in greater detail in Supplementary Note 1 and is informed by the previous work of refs. ^6,24–28^.

In Fig. 2b, we show the singlet return signal as a function of time spent at the manipulation point in (1,1) as the external magnetic field is varied along the $[1\bar 10]$ crystallographic direction. The observed oscillations demonstrate the ability to control coherent rotations. The rotation frequency displays a clear magnetic field dependence. In Fig. 2c, we plot the SO-induced rotation frequency as a function of field for both the [110] and $[1\bar 10]_{}^{}$ directions. The linear dependence on field is consistent with a *g*-factor difference between the two QDs (*f* = (Δ*g*)*μ*_B_*B*/*h*), whereas the difference in the slopes indicates an angular dependence for Δ*g*. We plot the full angular dependence of the SO interaction in Fig. 2d and e. Figure 2d shows the measured difference in gyromagnetic ratio between the dots, (Δ*g*)*μ*_B_/*h*, as a function of the in-plane angle *ϕ* relative to the [100] crystallographic direction. Dependence on the out-of-plane angle, *θ*, is shown in Fig. 2e. Here, *ϕ* is fixed along the [110] ([1$\bar 1$0]) direction and the measured difference in gyromagnetic ratio between the dots is plotted in blue (red) as the field is tilted out of the interface plane (*θ* = 0 is along the [001] direction). Qualitatively, the angular dependence is consistent with a SO effect, slightly different in each QD, composed of Rashba and Dresselhaus contributions. Enhanced interface–SO effects in Si have been surmised previously for in-plane magnetic field dependences^27,28,39–41^. We plot fits to Eq. (2) along with the data in Fig. 2d and e. We extract relative SO parameters Δ*α* = 1.89 MHz T^−1^ and Δ*β* = 15.7 MHz T^−1^. The maximum useful magnetic field is limited by state preparation and measurement (SPAM) errors as the *S* − *T*_−_ splitting becomes comparable to *k*_B_*T*. The maximum rotation frequency achieved for the present electrostatic confinement was near 20 MHz for fields above 1 T along the $[1\bar 10]$ direction.

**Fig. 3:**
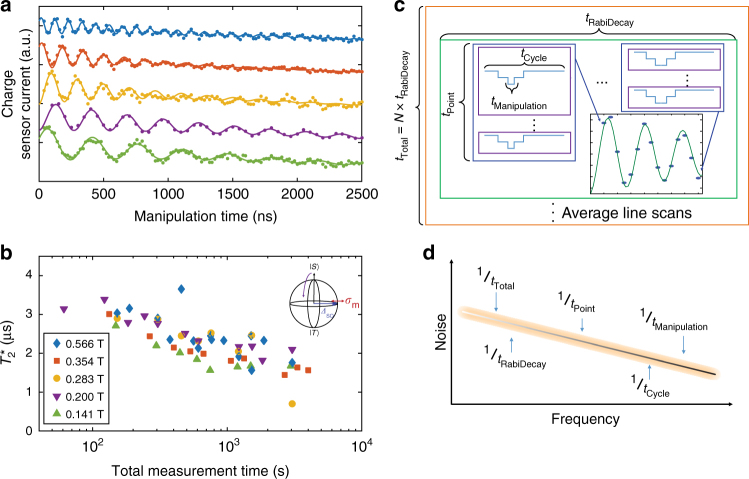
Measurement time dependence. **a** Long-time (50 min) averaged measurements of singlet return signal as a function of manipulation time for several magnetic field strengths aligned along the $[1\bar 10]$ crystallographic direction. The data for each field has been shifted for clarity. **b** The extracted $T_{\mathrm{2}}^ \star$ as a function of total experimental measurement time. (inset) Magnetic noise creates fluctuations in the effective magnetic field at each QD, leading to variation in the *X*-rotation frequency throughout the measurement. **c** Relevant time scales of the measurement. The shortest time scale susceptible to noise in the experiment is the time spent manipulating the qubit. In the limit of quasi-static noise, we expect the qubit to have a constant environment during this time. However, over the course of a total pulse cycle (which consists of qubit preparation and measurement and may be several ms in length), the environment may change. Furthermore, as the cycle is repeated and averaged by the lock-in for each data point, each data point is collected for a free induction decay curve. As successive curves are averaged together, the distribution of noise that is sampled grows larger. **d** During the course of the measurement, the qubit is susceptible to noise in the frequency band between 1/*t*_Total_ and 1/*t*_Manipulation_

The ability to realize meaningful quantum information processing in MOS depends on the timescale over which environmental noise near the interface interacts with the qubit, Fig. 3c and d. Although sparse, the background ^29^Si nuclear spins are sufficient in number to produce a slowly varying effective magnetic field, an Overhauser field. Nuclear spin flip-flops lead to a time-variation of the Overhauser field that is quasi-static on the timescale of a single measurement instance, but can shift the rotation frequency in the time interval between measurements. A consequence of this effect is that the decay in time of the coherent oscillations depends on the measurement integration time, as has been observed previously in ST qubits^12,42^. The longer an average measurement is done, the broader the distribution of spin configurations (i.e., Overhauser fields) sampled. The ensemble-averaged singlet return signal as a function of time spent driving rotations in the (1, 1) region, with an external magnetic field oriented along the $[1\bar 10]$ crystallographic axis, is shown in Fig. 3a. The decay in oscillation amplitude fits a Gaussian form consistent with quasi-static noise^42^, and characteristic inhomogeneous dephasing time, $T_{\mathrm{2}}^ \star$, is extracted assuming a functional time dependence of ${\mathrm{exp}}[ { - \left( {t{\mathrm{/}}T_{\mathrm{2}}^ \star } \right)^2} ]$ for the oscillation decay envelope.

In Fig. 3b, we examine the dependence of our results on measurement time and magnetic field. We find a long-averaging inhomogeneous dephasing time of $T_{\mathrm{2}}^ \star$ = 1.6 ± 0.6 μs, which is consistent within an order of magnitude with other DQD experimental results^12,19^ and theoretical estimates^43–45^ (see Supplementary Note 3) of the ergodic limit of the dephasing due to hyperfine coupling of the QD electron wave function with residual ^29^Si. By measuring at faster timescales, an increased $T_{\mathrm{2}}^ \star$ ~ 4 μs is observed. The absence of a magnetic field dependence suggests that the SO coupling does not contribute appreciably to $T_{\mathrm{2}}^ \star$. Therefore, the $T_{\mathrm{2}}^ \star$ observed at the MOS interface is consistent with expectations of the ^29^Si enriched bulk Si, and there is no evidence of additional noise due to the MOS interface at this enrichment level.

### Characterization of MOS charge noise

**Fig. 4:**
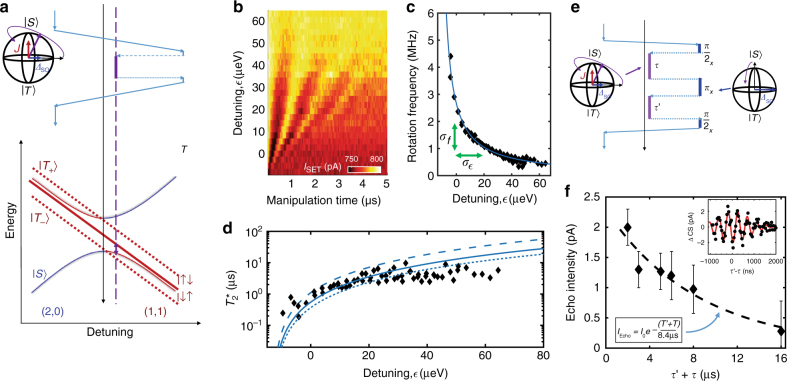
*Z-*rotations and noise. **a** Energy Diagram and gate pulse schematic for controlling exchange rotations. We initialize the qubit into the *S*(2, 0) ground state and ramp adiabatically, such that it transfers to the ground in the (1, 1) charge sector. A fast pulse to and from a detuning, $\epsilon$, where *J* is substantial drives coherent rotations around an axis depending on both *J* and *Δ*_SO_. Returning to the (2, 0) charge sector adiabatically projects the states onto the *S*(2, 0) and *T*_0_(1, 1) basis for measurement. **b** Measured charge sensor current as a function of the time spent rotating for various detuning points. Here, high current corresponds to a higher probability of measuring a singlet. **c** The extracted rotation frequency vs. detuning. The blue line is a fit to the form $\sqrt {J(\epsilon )^2 + {\mathit{\Delta }}_{{\mathrm{SO}}}^2}$, where *J*($\epsilon$) ∝ $\epsilon$^−1^. **d** Extracted $T_{\mathrm{2}}^ \star$ as a function of detuning. We also plot the long integration time values from Fig. 3a. The blue lines are fits to the form $T_{\mathrm{2}}^ \star$ = $\frac{1}{{\sqrt 2 \pi \sigma _e}} \cdot \left| {\frac{{{\mathrm{d}}f}}{{{\mathrm{d}}\epsilon }}} \right|^{ - 1}$, where the extracted charge noise, $\sigma _\epsilon$, is 1 (dashed), 2 (solid) and 3 μeV (dotted). **e** Gate pulse schematic for a Hahn-echo sequence. We initialize the qubit into the *S*(2, 0) ground state and transfer the system to the (1, 1) charge sector with a rapid adiabatic pulse such that it remains in a singlet state. Combinations of *Δ*_SO_-rotations about the *X*-axis and *J*-rotations about a second axis provide access to entire Bloch sphere. This echo sequence counteracts low frequency noise, prolonging qubit coherence. **f** Hahn-echo amplitude as a function of total time, *τ*′ + *τ*, exposed to charge noise at detuning $\epsilon$. The error bars represent 95% confidence interval. A fit to an exponential decay gives qubit coherence time of $T_{2e}^{{\mathrm{echo}}}$ = 8.4 μs. (inset) Measured echo signal for *τ* = 1 μs with B = 0.141 T along the [110] direction. The change in charge sensor current (ΔCS) due to the echo signal has an oscillation frequency corresponding to *Δ*_SO_ and a Gaussian envelope around *τ* = *τ*′ with a decay due to the inhomogeneous dephasing time of $T_{2e}^ \ast$ = 1 μs

A second axis of coherent control for ST qubits is achieved through the tunable exchange coupling of the (1, 1) and (2, 0) charge states. This leads to hybridization between the (2, 0) and (1, 1) charge states and an exchange splitting, *J*($\epsilon$), between the *S* and *T*_0_ qubit states that depends on detuning, $\epsilon$ (Fig. 4a). By varying the strength of this interaction, we can achieve controlled coherent rotations, as demonstrated in Fig. 4b. Here, as described in ref. ^46^, we initialize into a *S*(2, 0) ground state and then adiabatically separate the electrons into the (1, 1) charge configuration where *J*($\epsilon$) is nearly zero and the qubit is initialized in the ground state of the SO field ($\left| { \uparrow \downarrow } \right\rangle$ or $\left| { \downarrow \uparrow } \right\rangle$), a superposition of the *S*(1, 1) and *T*_0_(1, 1) states. We apply a fast pulse to and from finite *J*($\epsilon$) at $\epsilon$ near 0 for some waiting time, which rotates the qubit state around the Bloch sphere about a rotation axis depending on both *J* and *Δ*_SO_, the SO-induced splitting of the $\left| { \uparrow \downarrow } \right\rangle$ and $\left| { \downarrow \uparrow } \right\rangle$ states (Fig. 4a). For this experiment, we apply a field of 0.2 T along the [100] direction, which provides a small (0.5 MHz) residual *X*-rotation frequency. At detuning near $\epsilon$ = 0, we observe an increased rotation frequency (Fig. 4c). As the exchange pulse moves to deeper detuning, we observe a decrease in rotation frequency as well as visibility. This is expected as *J* decreases and the rotation axis tilts towards the direction of the SO field difference.

Figure 4c shows the observed rotation frequency as a function of detuning. The rotation frequency can be expressed as $\sqrt {J(\epsilon )^2 + {\mathit{\Delta }}_{{\mathrm{SO}}}^2}$, since the two components add in quadrature. Indeed, we see that at deep detuning the rotation frequency saturates near 0.5 MHz, due to the SO field at this magnetic field strength and orientation. Figure 4d shows the dephasing time, $T_{\mathrm{2}}^ \star$, associated with coherent rotations at each detuning. Here we have extracted $T_{\mathrm{2}}^ \star$ by fitting a Gaussian decay envelope $( {{\mathrm{exp}}[ { - ( {t{\mathrm{/}}T_{\mathrm{2}}^ \star } )^2} ]} )$ to the rotations at each detuning point. Noise from charge fluctuations on the confinement gates causes deviations in the detuning point of the system, leading to dephasing of the qubit through changes in the rotation frequency. We measure shorter dephasing time near $\epsilon$ = 0, which increases as we move to deeper detuning and eventually saturates at a few μs. We associate the saturation of $T_{\mathrm{2}}^ \star$ at deeper detuning with the dominant noise mechanism transitioning from charge to magnetic noise due to residual background ^29^Si. Following the method outlined in ref. ^42^, we fit the rotation frequency to a smooth function to find the derivative, d*f*($\epsilon$)/d$\epsilon$. The ratio of $T_{\mathrm{2}}^ \star$ to $\left| {{\mathrm{d}}f{\mathrm{/}}{\mathrm{d}}\epsilon } \right|^{ - 1}$ gives a root-mean-squared charge noise of *σ*_*ε*_ = 2 ± 0.6 μeV. This agreement with the best reported charge noise values in GaAs/AlGaAs and Si/SiGe material systems of a few μeV^16,18,19,42,47^ indicates that the poly-silicon MOS device structure is a competitive material system with respect to the magnitude of quasi-static charge noise. Furthermore, successive measurements over the course of several weeks can be performed with no retuning of the device gate voltages, indicating that the MOS material system is an extremely stable qubit platform.

Improved decoherence can be achieved through dynamical decoupling (DD), which suppresses contributions from quasi-static noise through multi-rotation sequences that leverage time reversal symmetry. A schematic for a Hahn-echo sequence to examine electrical noise is shown in Fig. 4e. As seen in Fig. 4f, a refocusing pulse can extend the qubit coherence with a $T_{{\mathrm{2e}}}^{{\mathrm{echo}}}$ of 8.4 μs for a detuning, $\epsilon$, where charge noise leads to $T_{{\mathrm{2e}}}^ \ast$ = 1 μs. This is comparable to what has been observed in GaAs/AlGaAs^42^ and Si/SiGe^12^. Likewise, Hahn-echo techniques were able to improve decoherence from magnetic noise to a $T_{{\mathrm{2m}}}^{{\mathrm{echo}}}$ of 70 μs (see Supplementary Note 3). These results illustrate our ability to extend coherence times through dynamical decoupling and unequivocally demonstrate full all-electrical control of the MOS SO-driven ST qubit.

## Discussion

In previous implementations of ST qubits, dynamic nuclear polarization (DNP)^48,49^, single nuclei^20,21^ and micro-magnets^19^ have been used to create strong, stable difference in Zeeman splitting between two quantum dots to drive rotations. The SO-driven *X*-rotations presented here reach 20 MHz and limited primarily by preparation and readout constraints (see Supplementary Note 3). Though this is larger than what has been reported for a ST qubit in Si/SiGe using a micromagnet^19^, it is smaller than the difference in Zeeman spin splitting of 50 to 1000 MHz between QDs reported in a number of other implementations mentioned above^20,49,50^. Increased drive frequency with SO coupling is likely possible through a number of avenues. Increasing the vertical electric field (see Supplementary Note 1 and ref. ^6^) and modifying the confinement potential (see Supplementary Note 1) will increase the strength of both the Rashba and Dresselhaus couplings. Additionally, the effect may be maximized by working with one of the QDs at higher occupation, since the two *z*-valleys at the hetero-interface are predicted to have opposite sign of the Dresselhaus strength (see SM and refs. ^6,27,28,30^). Single QDs have displayed a 140 MHz difference in ESR frequencies between electron occupations of *N* = 1 and *N* = 3 and electric field tunability^6^, so drive frequencies of over 100 MHz seem realistic.

On the other hand, our study of the angular dependence shows that by orienting the magnetic fields perpendicular to the interface, the difference in *g*-factor between the QDs is minimized. This is important for spin–qubit platforms where spin splitting variation is detrimental (e.g., spin-1/2 or exchange only qubits). This work also provides a theoretical foundation for the full angular dependence of an interface Dresselhaus and Rashba effect that avoids quantitative ambiguities due to gauge-dependence. Future work also remains to establish how the microscopic details of the MOS interface affects the magnitudes of the Rashba and Dresselhaus terms.

Most significantly from this work, the SO-driven ST qubit is a sensitive probe of noise properties at the MOS interface. The $T_{\mathrm{2}}^ \star$ of order 1–2 μs observed in the magnetic noise dominated regime is consistent with the ergodic limit expected from ^29^Si (i.e. order of magnitude agreement). Charge noise magnitudes of 2 ± 0.6 μeV at *T*_e_ ~ 150 mK are observed and are comparable to other semiconductor systems. Overall, the MOS interface shows no indication of increased negative effects relative to qubit operation despite the imperfect dielectric/crystal interface. The opportunity to use MOS for highly sensitive spin coherent devices such as qubits has broad impact. Considering the possibilities for improvement and the reduced complexity in fabrication, the SO-driven ST qubit offers a promising implementation for quantum information technology.

## Methods

### DQD device and experimental set-up

The DQD studied in this work was realized in a fully foundry-compatible (i.e. subtractive processing), single-gate-layer, isotopically enriched ^28^Si MOS device structure. The material stack consists of 200 nm highly Arsenic-doped (5 × 10^15^ cm^−2^ at 50 keV) poly-silicon and 35 nm of silicon-oxide on top of a silicon substrate with an isotopically enriched epitaxial layer hosting 500 ppm residual ^29^Si. Ohmic implants are formed using optical lithography and implantation of As at 3 × 10^15^ cm^−2^ at 100 keV. The confinement and depletion gates are defined by electron beam lithography followed by selective dry etching of the poly-silicon. Phosphorus donors were implanted (4 × 10^11^ cm^−2^ at 45 keV) through a self-aligned implant window near the QD locations for alternative experiments^20,21^. This was followed by an activation annealing process at 900 °C for 10 min in O_2_ and 5 min in N_2_ plus another 5 min in N_2_ at 1000 °C and a forming gas anneal at 400 °C.

Biasing the poly-silicon gates confines a 2-dimensional electron gas into quantum dot potentials. One QD is used as a single electron transistor (SET) remote charge sensor for spin-to-charge conversion. The rest of the device is tuned such that a DQD is formed, where one QD, define by the gate geometry, is tunnel coupled to a second, non-lithographic, QD formed nearby. This second QD, though unintended, survives thermal cycling and is a built-in feature of this device. The number of electrons in each QD is inferred from changes in current through the SET. Measurements were performed in a ^3^He/^4^He dilution refrigerator with a base temperature of around 8 mK. The effective electron temperature in the device was 150 mK. Fast RF lines we connected to cryogenic RC bias tees on the sample board, which to allow for the application of fast gate pulses. An external magnetic field was applied using a 3-axis vector magnet. Additional information discussing the device and measurements is offered in the Supplementary Material and elsewhere^5^.

### Data availability

The authors declare that the data supporting the findings of this study are available within the paper and its Supplementary Information files. Additional data (e.g., source data for figures) are available from the corresponding author upon reasonable request.

## Electronic supplementary material

Supplementary Information
