# Supplementary material for: A silicon metal-oxide-semiconductor electron spin-orbit qubit
Source: Nat Commun. 2018 May 2;9:1768. doi: 10.1038/s41467-018-04200-0 (PMC5931988; doi:10.1038/s41467-018-04200-0)
Supplement: Supplementary file 1 — Supplementary Information [file 41467_2018_4200_MOESM1_ESM.pdf]

SUPPLEMENTARY NOTE 1. Spin-orbit Coupling at the MOS Interface

While spin-orbit (SO) coupling in bulk silicon is weaker than in some other materials commonly used for quantum dot devices, such as GaAs and InAs, an interface introduces SO coupling that may significantly influence qubit operation. Such effects have been documented recently elsewhere in the case of a single quantum dot in silicon [1–6]. Here, we detail our model for the SO coupling associated with the interface. Our theoretical treatment is informed by the previous work of Refs [3, 7–9].

The Hamiltonian for a single electron in a silicon quantum dot in an arbitrary uniform magnetic field $\mathbf{B}$, without SO coupling included, is given by

$$\begin{aligned} H_0 &= H_{\text{dot}} + H_{\text{Zeeman}} \\ &= \frac{P_x^2}{2m_\perp} + \frac{P_y^2}{2m_\perp} + \frac{P_z^2}{2m_\parallel} + V(\mathbf{r}) + \frac{\mu_{\text{B}}}{2}\mathbf{B}\cdot\mathbf{g}_0\cdot\boldsymbol{\sigma}, \end{aligned} \tag{1}$$

where $\mathbf{P} = -i\hbar\nabla + e\mathbf{A}(\mathbf{r})$ is the kinetic momentum ($e > 0$), $m_\perp = 0.19m_0$ ($m_\parallel = 0.98m_0$) is the transverse (longitudinal) effective mass, and $\mathbf{g}_0 = \text{diag}(g_\perp, g_\perp, g_\parallel)$ is the bulk g-tensor for silicon. We take our coordinate system to be aligned along the Cartesian [100], [010], [001] axes, with [001] the interface normal. The potential $V(\mathbf{r})$ includes electrostatic confinement from voltages applied to gate electrodes and details of the interface potential. Atomic-scale features at the interface, the potential barrier height, and the vertical electric field dictate the valley splitting and valley content of the valley-orbital eigenstates [10]. Due to the strong vertical confinement, the low-lying valley-orbital eigenstates include contributions only from the $\pm z$ conduction band minima. As a consequence of the weak bulk SO coupling in silicon, $g_\perp$ and $g_\parallel$ are close to the vacuum g-factor of 2.0. In our double quantum dot device the bulk g-factor anisotropy, being common to both dots, does not manifest in significant measurable effects. The specific gauge choice for $\mathbf{A}$ has no influence on any physical observables, and we emphasize that any theoretical analysis must be gauge-invariant. When necessary for numerical calculations, we choose the convenient gauge $\mathbf{A}(\mathbf{r}) = \frac{1}{2}\mathbf{B}\times\mathbf{r}$.

Assuming that we have found the valley-orbital eigenstates of the spin-independent part of H_0, H_{dot}, we now treat the SO coupling as a perturbation. Following Refs [3, 7–9], we take the SO interaction for an electron confined against an interface at $z = z_i$ to consist of both Rashba and Dresselhaus terms, $H_R = \gamma_R\delta(z - z_i)(P_y\sigma_x - P_x\sigma_y)$ and $H_D = \gamma_D\delta(z - z_i)(P_x\sigma_x - P_y\sigma_y)$, respectively [11]. We emphasize the importance of the interface-localized δ-function in these terms. As we will see, this leads to the SO coupling appearing at first order in perturbation theory, rather than second order if the SO coupling had taken the bulk form without the interface-localizing δ-function. This latter property can be seen from the fact that, for bound valley-orbital eigenstates $|v_k\rangle$, the diagonal momentum matrix elements vanish, $\langle v_k|\mathbf{P}|v_k\rangle = 0$, without approximation. This can be confirmed by applying the commutation identities $P_x = \frac{im_\perp}{\hbar}[H_0, x]$, $P_y = \frac{im_\perp}{\hbar}[H_0, y]$, and $P_z = \frac{im_\parallel}{\hbar}[H_0, z]$. However, the interface-constrained diagonal matrix elements $\langle v_k|\delta(z - z_i)\mathbf{P}|v_k\rangle$ may be non-zero, in general, due to the cyclotron orbits established by an applied magnetic field. Note that, due to the crystal symmetry of silicon, a vertical shift of a pristine (001) interface by $z \to z + a_0/4$, where $a_0 = 0.543$ nm is the lattice constant, is equivalent to an in-plane rotation by an angle $\pi/2$. Consequently, while a Rashba term $P_y\sigma_x - P_x\sigma_y$ is invariant, a Dresselhaus term $P_x\sigma_x - P_y\sigma_y$ must change sign under such a transformation[8], since a $\pi/2$ rotation maps $P_y \to P_x$, $P_x \to -P_y$, $\sigma_y \to \sigma_x$, and $\sigma_x \to -\sigma_y$. Hence, we assign the Dresselhaus coupling factor a dependence $\gamma_D(z_i) = \gamma_D\cos(4\pi z_i/a_0)$ to capture the rapidly oscillatory behavior of the sign of the Dresselhaus coupling as a function of interface position.

To proceed with identifying the contributions of H_{SO}, we must evaluate the interface-constrained momentum matrix elements $\langle v_k|\delta(z - z_i)\mathbf{P}|v_j\rangle$ as a function of the applied magnetic field, $\mathbf{B}$. We note that the effective SO coupling strengths γ_R and γ_D should be expected to depend intimately on the atomistic details of the interface [7–9]. For the purposes of this analysis, we wrap such details into Rashba and Dresselhaus coupling strengths α_R and β_D, respectively, and treat them as fit parameters. Future work will address the question of capturing the short length-scale physics of interface SO effects within a multi-valley effective mass theory framework[12], in the spirit of previous analyses of valley splitting statistics in the presence of interface disorder[10].

The valley composition of the valley-orbital eigenstates $|v_k\rangle$ is dictated by the relative phase between the $+z$ and $-z$ valley components. Within a simplified envelope function picture (see e.g. Ref. [13]), the low-lying valley components are given by

$$|+z\rangle = e^{ik_0z}u_{+z}(\mathbf{r})\psi(\mathbf{r}) \tag{2}$$

$$|-z\rangle = e^{-ik_0z}u_{-z}(\mathbf{r})\psi(\mathbf{r}), \tag{3}$$

where $k_0 = 0.84\pi/a_0$ is the position of the conduction band minimum, $u_{\pm z}(\mathbf{r})$ are the lattice-commensurate Bloch functions for silicon's $\pm z$ conduction band minima, and $\psi(\mathbf{r})$ is an envelope function. The lowest two valley-orbital

eigenstates are, then

$$|v_0\rangle = \frac{1}{\sqrt{2}}(|{+z}\rangle + e^{i\phi_{\mathrm{v}}}|{-z}\rangle) \tag{4}$$

$$|v_1\rangle = \frac{1}{\sqrt{2}}(|{+z}\rangle - e^{i\phi_{\mathrm{v}}}|{-z}\rangle), \tag{5}$$

where ϕ_{v} is the valley phase factor. As mentioned previously, the value of ϕ_{v} and the valley splitting $\Delta_{\mathrm{vs}} = \langle v_1|H_{\mathrm{dot}}|v_1\rangle - \langle v_0|H_{\mathrm{dot}}|v_0\rangle$ is dictated by details of the interface and associated confinement potential.

In particular, we approximate

$$\langle v_0|\delta(z-z_i)P_j|v_0\rangle = \frac{c}{2}\left(1+\cos\left(\phi_{\mathrm{v}}-2k_0z_i\right)\right)\langle\psi|\delta(z-z_i)P_j|\psi\rangle, \tag{6}$$

where

$$\langle\psi|\delta(z-z_i)P_j|\psi\rangle = \iint \mathrm{d}x\mathrm{d}y\ \psi^*(x,y,z_i)P_j\psi(x,y,z_i), \tag{7}$$

with $\psi(\mathbf{r})$ the envelope function and c an unknown real parameter that depends on details of the Bloch function at the interface. Similarly, for the first excited valley state we'd obtain

$$\langle v_1|\delta(z-z_i)P_j|v_1\rangle = \frac{c}{2}\left(1-\cos\left(\phi_{\mathrm{v}}-2k_0z_i\right)\right)\langle\psi|\delta(z-z_i)P_j|\psi\rangle. \tag{8}$$

To investigate the momentum matrix element $\langle\psi|\delta(z-z_i)P_j|\psi\rangle$ with respect to the envelope function, we have implemented a (valley-free) finite-difference discretization of a Hamiltonian for a quantum dot that is harmonically confined laterally, with a uniform vertical electric field F_z and an interface with energy offset U_0,

$$\begin{aligned} H = &\frac{P_x^2}{2m_\perp} + \frac{P_y^2}{2m_\perp} + \frac{P_z^2}{2m_\parallel} \\ &+\frac{1}{2}m_\perp\omega_x^2x^2 + \frac{1}{2}m_\perp\omega_y^2y^2 + F_zz + U_0\Theta(z) \end{aligned} \tag{9}$$

The harmonic confinement energies $\hbar\omega_x$, $\hbar\omega_y$ are allowed to be distinct, describing an anisotropically-shaped quantum dot. From qualitative fits to a numerical analysis, we find the following functional form for the matrix elements with respect to the ground state envelope function:

$$\langle\psi|\delta(z-z_i)P_x|\psi\rangle \approx (a - b\hbar\omega_x)F_z^{2/3}B_y \tag{10}$$

$$\langle\psi|\delta(z-z_i)P_y|\psi\rangle \approx -(a - b\hbar\omega_y)F_z^{2/3}B_x, \tag{11}$$

where for dot confinement energies of $\mathcal{O}(\mathrm{meV})$ we find $(b \times 1\ \mathrm{meV})/a \approx 2\%$. The $F_z^{2/3}$ dependence is consistent with what is expected for a triangular vertical confinement potential [3]. Notice that these matrix elements depend weakly on the lateral confinement energies, with the dominant dependence on the vertical electric field and transverse magnetic field. This qualitative functional dependence on magnetic and vertical electric fields is consistent with the analysis of Ref [3]. In Supplementary Figure 1, we plot a representative momentum density, indicating the cyclotron orbits induced by the applied magnetic field.

Combining the envelope function and valley components together, we obtain the following functional form for the interface-constrained momentum matrix elements:

$$\begin{aligned} \langle v_0|\delta(z-z_i)P_x|v_0\rangle &\propto (1+\cos\left(\phi_{\mathrm{v}}-2k_0z_i\right)) \\ &\quad\times(a - b\hbar\omega_x)F_z^{2/3}B_y \\ &= \lambda(\phi_{\mathrm{v}}, z_i, F_z, \hbar\omega_x)B_y \end{aligned} \tag{12}$$

$$\begin{aligned} \langle v_0|\delta(z-z_i)P_y|v_0\rangle &\propto -(1+\cos\left(\phi_{\mathrm{v}}-2k_0z_i\right)) \\ &\quad\times(a - b\hbar\omega_y)F_z^{2/3}B_x \\ &= -\lambda(\phi_{\mathrm{v}}, z_i, F_z, \hbar\omega_y)B_x, \end{aligned} \tag{13}$$

where $\lambda(\phi_{\mathrm{v}}, z_i, F_z, \hbar\omega)$ is a function that encodes the dependence on valley phase, interface location, vertical electric field, and lateral confinement.

While the SO interaction will induce non-zero matrix elements between valley-orbital eigenstates such as $\langle v_0\uparrow$

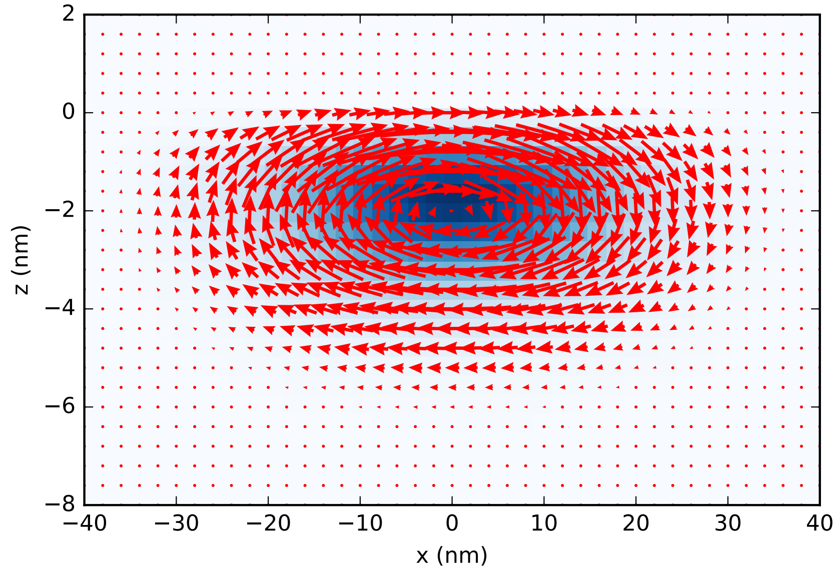

Supplementary Figure 1. Quantum dot wave function. Kinetic momentum density and cyclotron motion of the ground state of a quantum dot with an applied magnetic field $\mathbf{B} = B\hat{\mathbf{y}}$, for a slice at $y = 0$. In blue we show the probability density of the electron. The interface with offset $U_0 = 3$ eV is located at $z = 0$, vertical field is $F_z = 10$ MV m^{-1}, and lateral confinement energies are $\hbar\omega_x = 1$ meV, $\hbar\omega_y = 3$ meV. Notice that $\langle\psi|P_x|\psi\rangle = 0$, while $\langle\psi|\delta(z)P_x|\psi\rangle > 0$.

$|H_{\rm SO}|v_1\downarrow\rangle$, the influence of these matrix elements will be suppressed by the valley splitting $\Delta_{\rm vs}$ [3]. Since valley splitting in MOS systems is typically relatively large ($\mathcal{O}(100$ µeV) in this experiment) and these inter-valley eigenstate matrix elements appear to second order in perturbation theory, we neglect them here.

Within the subspace spanned by the tensor product of the lowest valley-orbital eigenstate $|v_0\rangle$ and σ_z spin eigenstates $|\uparrow\rangle$, $|\downarrow\rangle$, $\{|v_0\uparrow\rangle, |v_0\downarrow\rangle$, we can express the SO Hamiltonian as

$$H_{\rm SO} = (-\gamma_R\lambda_y B_x + \gamma_D\lambda_x B_y)\sigma_x + (-\gamma_R\lambda_x B_y + \gamma_D\lambda_y)\sigma_y. \tag{14}$$

If we make the approximation that the dot is nearly symmetric, $\omega_x \approx \omega_y$, then this reduces to the form

$$\begin{aligned} H_{\rm SO} &= (-\alpha_R B_x + \beta_D B_y)\sigma_x + (-\alpha_R B_y + \beta_D B_x)\sigma_y \\ &= \frac{\mu_{\rm B}}{2}\mathbf{B}\cdot\mathbf{g}_{\rm SO}\cdot\boldsymbol{\sigma}, \end{aligned} \tag{15}$$

where

$$\mathbf{g}_{\rm SO} = \frac{2}{\mu_{\rm B}}\begin{pmatrix} -\alpha_R & \beta_D & 0 \\ \beta_D & -\alpha_R & 0 \\ 0 & 0 & 0 \end{pmatrix}. \tag{16}$$

Since we expect $a >> b\hbar\omega_{x,y}$, a non circular dot would include only small corrections to this form. Consequently, the interface SO interaction in a quantum dot can be represented as a modified g-tensor $\mathbf{g} = \mathbf{g}_0 + \mathbf{g}_{\rm SO}$, where

$$\mathbf{g} = \begin{pmatrix} g_\perp - 2\alpha_R/\mu_{\rm B} & 2\beta_D/\mu_{\rm B} & 0 \\ 2\beta_D/\mu_{\rm B} & g_\perp - 2\alpha_R/\mu_{\rm B} & 0 \\ 0 & 0 & g_\parallel \end{pmatrix}. \tag{17}$$

Note here that the total g-tensor $\mathbf{g}$ need not be symmetric, since any asymmetry in the quantum dot geometry may result in $g_{xy} \neq g_{yx}$, in general. However, in our fitting to the present experimental data we have observed satisfactory agreement when assuming a symmetric g-tensor. Future measurements with reduced statistical uncertainty or more anisotropic dot geometries may allow for this effect to be probed.

In the regime of deep detuning, for which the two electrons in the DQD are well delocalized into the $(1,1)$ charge configuration, we can treat the interface SO coupling as producing a distinct effective g-tensor in the left and right dots, $\mathbf{g}_L$ and $\mathbf{g}_R$. That is, the SO Hamiltonian transforms the Zeeman Hamiltonian for the two-electron problem into

$$H_{\mathrm{Zeeman}} = \frac{\mu_{\mathrm{B}}}{2}\mathbf{B}\cdot\mathbf{g}_L\cdot\boldsymbol{\sigma}_L + \frac{\mu_{\mathrm{B}}}{2}\mathbf{B}\cdot\mathbf{g}_R\cdot\boldsymbol{\sigma}_R, \tag{18}$$

where $\boldsymbol{\sigma}_L$ ($\boldsymbol{\sigma}_R$) is the vector of Pauli operators acting on an electron in the left (right) quantum dot.

We now show how this g-tensor difference appears in terms of the basis states $\{|S(1,1)\rangle, |T_+(1,1)\rangle, |T_0(1,1)\rangle, |T_-(1,1)\rangle\}$, where we follow the convention of Ref. [14]:

$$\begin{aligned} |S(1,1)\rangle &= \frac{1}{\sqrt{2}}\left(c^\dagger_{L\uparrow}c^\dagger_{R\downarrow} - c^\dagger_{L\downarrow}c^\dagger_{R\uparrow}\right)|\emptyset\rangle \\ |T_+(1,1)\rangle &= c^\dagger_{L\uparrow}c^\dagger_{R\uparrow}|\emptyset\rangle \\ |T_0(1,1)\rangle &= \frac{1}{\sqrt{2}}\left(c^\dagger_{L\uparrow}c^\dagger_{R\downarrow} + c^\dagger_{L\downarrow}c^\dagger_{R\uparrow}\right)|\emptyset\rangle \\ |T_-(1,1)\rangle &= c^\dagger_{L\downarrow}c^\dagger_{R\downarrow}|\emptyset\rangle, \end{aligned} \tag{19}$$

where $c^\dagger_{L\uparrow}$ ($c^\dagger_{R\uparrow}$) creates an electron in the left (right) quantum dot with spin up in the eigenbasis of σ_z (relative to the crystallographic axis [001]) and $|\emptyset\rangle$ is the zero-electron state. Given this set of basis states and defining

$$\begin{aligned} \delta\mathbf{b} &= \frac{\mu_{\mathrm{B}}}{2}\mathbf{B}\cdot(\mathbf{g}_L - \mathbf{g}_R)/2 \\ \bar{\mathbf{b}} &= \frac{\mu_{\mathrm{B}}}{2}\mathbf{B}\cdot(\mathbf{g}_L + \mathbf{g}_R)/2, \end{aligned} \tag{20}$$

we can now write down the Zeeman Hamiltonian incorporating SO coupling:

$$H_{\mathrm{Z}} = \begin{pmatrix} 0 & -\sqrt{2}(\delta b_x + i\delta b_y) & 2\delta b_z & \sqrt{2}(\delta b_x - i\delta b_y) \\ \cdot & 2\bar{b}_z & \sqrt{2}(\bar{b}_x - i\bar{b}_y) & 0 \\ \cdot & \cdot & 0 & \sqrt{2}(\bar{b}_x - i\bar{b}_y) \\ \cdot & \cdot & \cdot & -2\bar{b}_z \end{pmatrix},$$

where

$$\delta b_x = \frac{1}{2}(-B_x\Delta\alpha + B_y\Delta\beta) \tag{21}$$

$$\delta b_y = \frac{1}{2}(-B_y\Delta\alpha + B_x\Delta\beta) \tag{22}$$

$$\delta b_z = 0. \tag{23}$$

We now evaluate the unpolarized triplet spin eigenstate $|\tilde{T}_0(1,1)\rangle$ relative to the quantization axis dictated by the applied magnetic field, $\mathbf{B}$. Using the fact that the g-tensor is only weakly perturbed from its bulk value, $|\mathbf{g} - 2I| \ll 1$, and diagonalizing the 3x3 triplet block, we obtain

$$|\tilde{T}_0\rangle = \cos\theta|T_0\rangle + \frac{1}{\sqrt{2}}\sin\theta(e^{i\phi}|T_-\rangle - e^{-i\phi}|T_+\rangle), \tag{24}$$

where the applied magnetic field is taken to be

$$\mathbf{B} = |\mathbf{B}|(\sin\theta\cos\phi, \sin\theta\sin\phi, \cos\theta)) \tag{25}$$

with respect to the crystallographic axes [100], [010], and [001].

Finally, to evaluate the frequency of S/T_0 rotations generated by such a difference in g-tensors, we need to evaluate the matrix element $\langle S|H_{\mathrm{SO}}|\tilde{T}_0\rangle$, where $|S\rangle$ and $|\tilde{T}_0\rangle$ are the singlet and unpolarized triplet states defined with respect

to the spin basis of the applied uniform magnetic field $\mathbf{B}$ defined above. We find that this rotation frequency is

$$f_{\rm rot} = \frac{2}{h}|\langle S|H_{\rm SO}|\tilde{T}_0\rangle| \tag{26}$$

$$= \frac{4}{h}|\sin\theta\big(\cos\phi\delta b_x + 2\sin\phi\delta b_y\big)| \tag{27}$$

$$= \frac{2}{h}|\mathbf{B}||\Delta\alpha - \Delta\beta\sin(2\phi)|\sin^2\theta. \tag{28}$$

From the above expression, it's clear that applying a magnetic field normal to the interface $\theta = 0$ will generate no effective magnetic field gradient. For an in-plane field, depending on the relative sign of the Rashba and Dresselhaus differences $\Delta\alpha$ and $\Delta\beta$, there will be an azimuthal angle ϕ that maximizes the generated S/T_0 rotation frequency. If sign($\Delta\alpha$) = sign($\Delta\beta$) (sign($\Delta\alpha$) $\neq$ sign($\Delta\beta$)), the maximum rotation frequency will be obtained for $\phi = -\pi/4$ ($\phi = \pi/4$), i.e. magnetic field oriented along [1$\bar{1}$0] ([110]). Conversely, for $|\Delta\beta| \gg |\Delta\alpha|$ the minimum rotation frequency would be obtained for $\phi \approx 0$ or $\pi/2$, i.e. nearly aligned along the [100] or [010] Cartesian axes. In our experiment, with $|\Delta\beta/\Delta\alpha| \approx 8.3$, the minimum frequency should be attained with a magnetic field about 3.5 degrees away from the [100] orientation.

SUPPLEMENTARY NOTE 2. Device Fabrication, Structure, Operation

Device Structure

The singlet-triplet (ST) qubit studied in this work was fabricated in a fully foundry-compatible process using a single-gate-layer, metal-oxide-semiconductor (MOS) poly-silicon gate stack with an epitaxially-enriched ^{28}Si epi-layer with 500ppm residual ^{29}Si. Hall bars from the same sample wafer with the same gate oxide were used to extract the critical density ($n_{\rm c}$ = 5.7x10^{11} cm^{-2}), the peak mobility (μ =4500 cm^2 V^{-1} s^{-1}), threshold voltage (V_{th} = 1.1 V), the RMS interface roughness ($\mathit{\Delta}$ = 2.4 Å), and roughness correlation length (λ = 26 Å). An SEM image of a device fabricated nominally identically to the one used in this work and a schematic of the gate stack are shown in Supplementary Figure 2(a,b). The device is operated in an enhancement mode using voltage biasing of the highly doped n+ poly-silicon gates to confine electrons to quantum dot (QD) potentials under gates LCP and UCP. The gates ULG, URG, LLG and LRG overlap with n+ regions and ohmic contacts and are biased to accumulate a two-dimensional electron gas (2DEG) under each gate. The 2DEGs act as source and drain electron reservoirs for the quantum dots. The lower half of the device is tuned such that a double quantum dot (DQD) is formed. One QD is tunnel coupled to the reservoir under LRG and the other quantum dot can only be occupied by electron tunneling through the first QD. The upper half of the device is used as a single electron transistor (SET) remote charge sensor. The SET is biased with 70 μV (rms) AC bias at 0V DC and the current is measured with an AC lock-in technique at 979 Hz. The electron temperature, $T_{\rm e} \sim$ 150 mK, was measured by QD charge transition line width. More details about fabrication can be located in Ref [15].

Dot Occupation and Location

The number of electrons in each QD may be inferred from changes in current through the SET as depicted in Supplementary Figure 2(c). The collection of yellow parallel lines is assigned to a QD connected to the electron reservoir under LRG, which we call QD$_1$. Counting from the left, we can identify the QD$_1$ N=1 $\rightarrow$ N=2 charging transition. A second object is observed anti-crossing with QD$_1$, which we label as the N=0 $\rightarrow$ N=1 charge transition for a second QD, QD$_2$. A second line is observed belonging to QD$_2$ in the scan, though disorder in the system makes identifying higher occupation lines difficult. However, the presence of Pauli spin blockade at the QD$_1$-QD$_2$, (2,0)-(1,1) anti-crossing identifies the system as a useful DQD for a ST qubit (see Supplementary Figure 3). To determine the locations of QD$_1$ and QD$_2$ we can use their capacitances to the nearby poly-silicon gates. By scanning combinations of the poly-silicon gates (as seen in Supplementary Figure 2(c) for LLP and LCP), we can obtain the relative capacitance of both QDs to each gate compared to the capacitance of LCP, which has the strongest capacitive coupling to both QDs. We have tabulated the relative capacitances in Supplementary Table **??**. These values allow for triangulation of the dot locations, which we have indicated in S2(b) with open circles. We differentiate QD$_2$ from an implanted donor through several observations: (1) no hyperfine component in the rotation frequency, (2) the lack of rotations at 0 T magnetic field, (3) the ramp rates required for adiabatic transfer through the spin gap are slower than what is expected for a donor, and (4) the presence of additional lines corresponding to the QD. We find that this layout

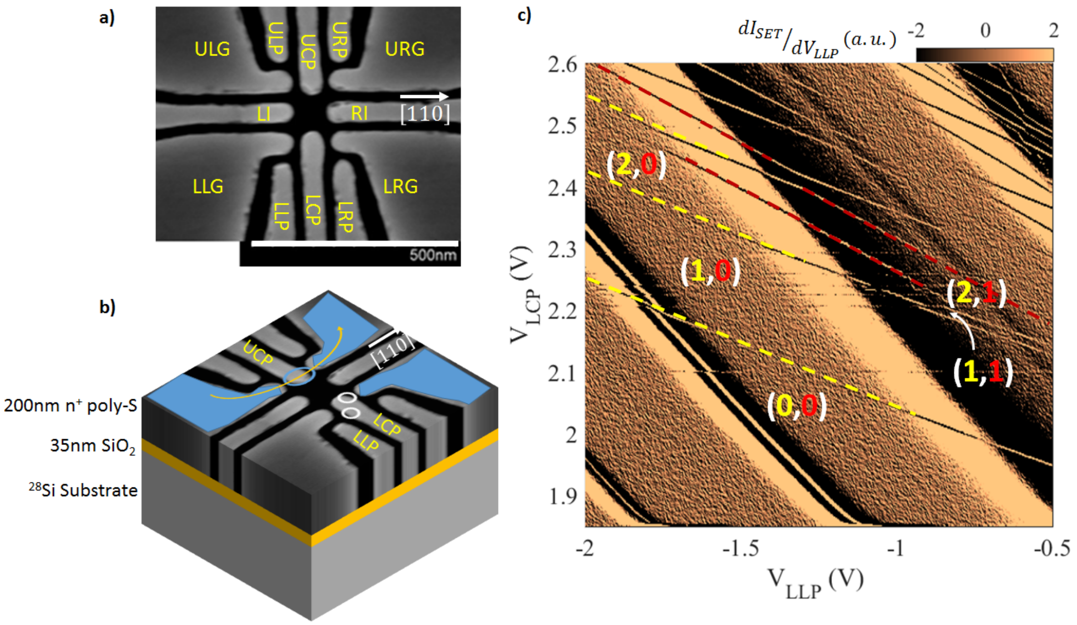

Supplementary Figure 2. Device Structure. a) A top-down SEM of the single-layer poly-silicon gate design. The gates are labeled in yellow and the [110] crystallographic direction is indicated in white. b) A cartoon schematic of the MOS gate-stack. The 2DEG reservoirs used in these experiments are illustrated in blue with the current through the top QD SET charge sensor depicted by the yellow arrow. The approximate locations of the two QDs are represented by the white circles. c) A charge stability diagram of the DQD. Here, the gradient of the current running through the SET charge sensor is plotted as the gates LLP and LCP are varied. The broad diagonal background features are due to Coulomb peaks of the SET charge sensor. The sharp features correspond to charge transitions of objects in the lower half of the device. QD_1 (the QD closer to the electron reservoir under gate LRG) is indicated by the successive yellow dashed lines, and QD_2 is indicated by the dashed red lines. The regions in gate space corresponding to different DQD charge occupations are labeled in parentheses with the occupation of QD_1 in yellow and QD_2 in red.

Supplementary Table 1. Gate capacitance to QDs relative to LCP

c_i/c_{LCP}	LLP	LRP	LI	RI	LLG	LRG
QD_1	0.18	0.33	0.24	0.16	0.29	0.35
QD_2	0.27	0.22	0.13	0.14	0.29	0.10

systematically produces objects near the central QD with these capacitances when the gates opposite the electron reservoir 2DEG are at low biases. For experiments investigating single QDs, these voltage potential minima may be emptied with more negative voltages on LLP or flooded by accumulating a larger 2DEG under LLG with more positive voltages.

Qubit Initialization, Operation and Readout

We operate this system near the (2,0) $\rightarrow$ (1,1) spin-blockaded (N_{QD_1},N_{QD_2}) charge anti-crossing. An energy diagram for the two-electron system is shown in Supplementary Figure 3(b). The ground state charge configuration is determined by the detuning between dots, ϵ, which is controlled by tuning the voltages on gates LLP and LCP. These gates are connected to cryogenic RC bias-Ts which allow the application of fast gate pulses. A schematic of the cyclical pulse sequence is shown in Supplementary Figure 3(a), which is repeated as the current through the SET is monitored by the averaged AC lock-in measurements. The system is initialized in the (2,0) charge sector by first unloading (point U) the DQD into the (1,0) charge configuration and then applying an energy-selective pulse into the

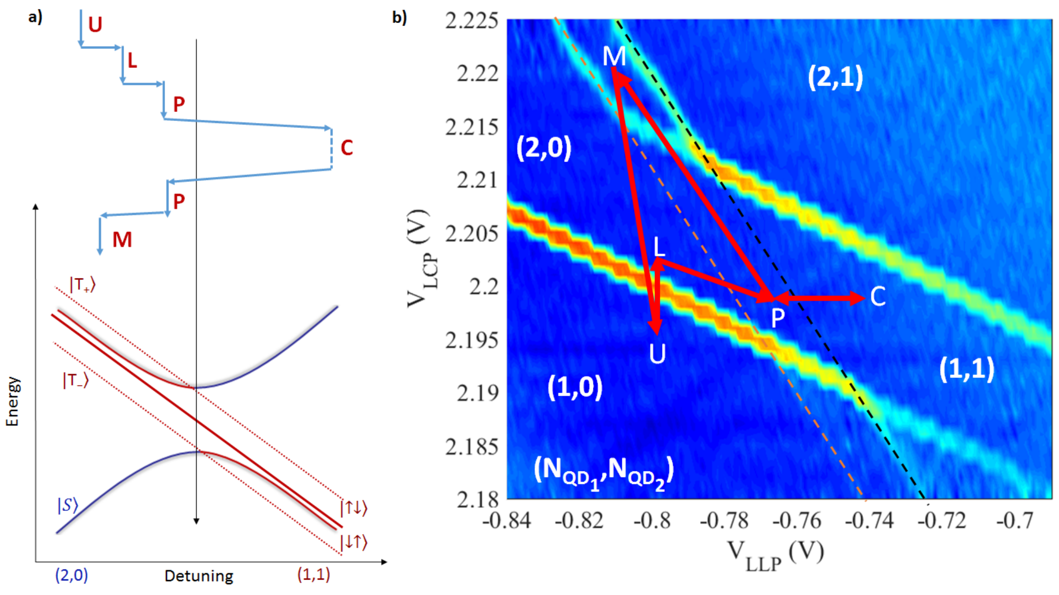

Supplementary Figure 3. Qubit Operation. a) Energy diagram and gate pulse schematic for qubit operations. b) A pulsed charge stability diagram for the (2,0) → (1,1) anticrossing, showing the gradient of the charge sensor current. The red arrows depict a general pulse sequence for controlling the qubit, where point C may consist of several detuning pulses for different qubit manipulation sequences. The black and orange dashed lines correspond to the location of the singlet and triplet state charge preserving lines, respectively. We do not observe a change in charge sensor current at the charge preserving lines due to the orientation of the DQD dipole.

(2,0) charge state between the singlet and triplet energy levels such that a (2,0)S ground state is loaded (point L). The system is then plunged (point P) to a detuning ($\epsilon < 0$) close to the charge anti-crossing. The electrons are then separated (point C) and qubit manipulations are performed in the (1,1) charge region ($\epsilon > 0$). The system is then pulsed back to the (2,0) charge sector (point P) where, due to Pauli spin blockade, a singlet spin state is allowed to transfer to the (2,0) charge state, but a triplet spin state is energetically blocked and remains as a (1,1) charge state [16]. We then use an enhanced latching mechanism for a spin-to-charge conversion (pulsing to point M), where the triplet state is mapped to a (2,1) charge state. and the singlet is read out as (2,0). This technique relies on a slow tunnel rate from QD_2 to a charge reservoir. This causes singlet states to remain locked in a metastable (2,0) charge state when pulsing the system to point M, as a slow co-tunneling process is required to equilibrate. On the other hand, triplet states may quickly transfer to (2,1) by inelastically tunneling an electron onto QD_1 from the lead. There are several advantages for the use of this method. First, since an electron on the QD_2 needs to tunnel through the QD_1 to access an electron reservoir, the metastable latching state can be long lived. This allows for the measurement step in our cyclical pulse sequence to be long, compared to other points in the sequence, and dominate the time average. Second, in this approach, the charge-sensed signal differentiates between a (2,1) and a (2,0) charge state. In other words, the difference in measured current between a singlet and triplet state is the capacitive effect of adding an electron to QD_2. Thus, it does not rely on the dipole orientation of the DQD, as in traditional Pauli-blockade measurement techniques. In our case, the DQD is oriented in such a way that differentiating a (2,0) and (1,1) charge state is exceptionally difficult (observe the lack of visible inter-dot transition line in Supplementary Figure 3(b)) and this method is necessary. This technique is presented in greater detail in [17]. Additionally, the latching effect has been used to take advantage of the enhanced spin-to-charge signal and lifetimes in a variety of QD-QD[18–21], donor-donor[22] and QD-donor[17, 23] coupled systems.

SUPPLEMENTARY NOTE 3. Analysis Note on Extracted Data

Qubit Rotation Frequency

Supplementary Figure 4(a) shows the singlet return signal as a function of time spent at the manipulation point in $(1,1)$ as the strength of the external magnetic field is varied along the $[1\bar{1}0]$ crystallographic direction up to 1.2 T.

We see that, at high magnetic field, the oscillations are difficult to observe, since the Coulomb blockade peak used for charge sensing drifts as a function of magnetic field. The qubit rotation frequency at each field was found by fitting each line scan to a Gaussian decay of the form

$$I_{SET} = A\sin(2\pi ft + \phi_0)\exp[-(t/T_2^\star)^2] + Bt + C \tag{29}$$

where all parameters are free. To help with the visualization, we subtract the background linear portion to our charge sensor signal ($Bt + C$, above), as shown in Supplementary Figure 4(b).The background slope in charge sensor current is due to imperfectly separating the two electrons, such that, for some fraction of the experiments, an electron diabatically transitions through the ani-crossing, thus inelastically transferring between S(2,0) and S(1,1) on the time scale of a few µs. The rotation frequency, f, corresponding to the data in Supplementary Figure 4(a) is plotted as a function of magnetic field in Supplementary Figure 4c, indicating a 20 MHz rotation frequency at the maximum field. As can be seen in Supplementary Figure 3(c), there are outlier points, which occur when a fit to equation (29), produces an unphysical periodic component. Similar techniques were used to analyze the data in the main text. The

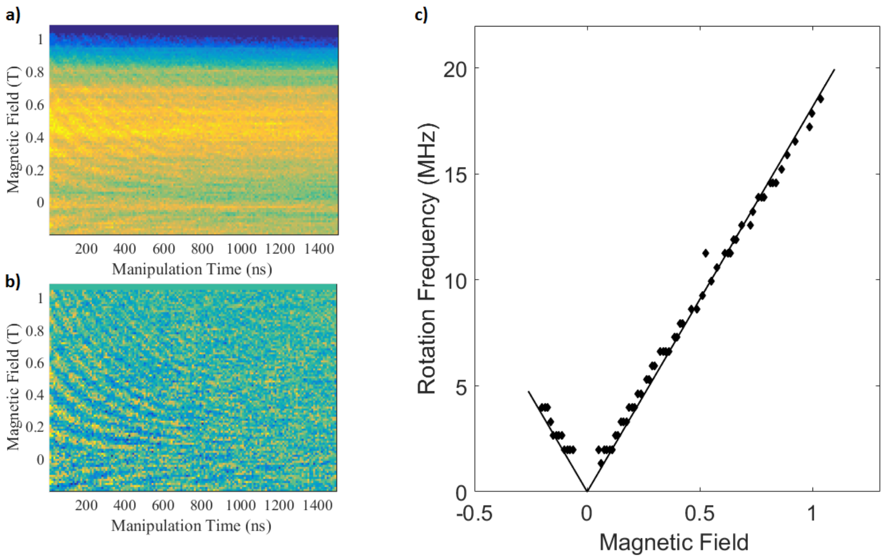

Supplementary Figure 4. Qubit Rotation Frequency Scan. a) Measured charge sensor current as a function of manipulation time as magnetic field is stepped. b) The measured data after subtracting the linear charge sensor background current. c) The extracted rotation frequency as a function of magnetic field. The solid line is a linear fit to magnetic field strength.

data presented in Figs. 2(c,e) of the main text were also obtained from magnetic field scan experiments and similar behavior was observed. A majority of the data fits well, and clear magnetic field strength and angular dependencies may be extracted. The data presented in Fig. 2(d), was taken with repeated scans at a given field strength and orientation. For Fig. 2(b), the linear portion of the background charge current sensor was subtracted, to clearly show the oscillations. This was useful, as slow timescale changes in the current through the charge sensor obscured the visualization.

Charge Noise Characterization

Here we describe the procedure to extract charge noise, following Ref [24]. Several results in ST qubits have shown that a dominant source of dephasing during exchange oscillations can be modeled as gate-referred, quasi-static voltage fluctuations on nearby gates[24, 25]. These voltage fluctuations affect the energy detuning between dots, materializing as fluctuations in the exchange energy, J. At a given detuning, the qubit will rotate at a frequency about the Bloch sphere

$$f(\epsilon) = \frac{1}{h}\sqrt{J(\epsilon)^2 + \Delta_{\mathrm{SO}}^2}. \tag{30}$$

Therefore, we expect noise in detuning $\delta\epsilon$ to create noise in the rotation frequency $\delta f \sim \delta\epsilon \cdot \mathrm{d}f/\mathrm{d}\epsilon$. For charge noise that is quasi-static, we expect a Gaussian decay of the oscillations of the form $\exp[-(t/T_2^\star)^2]$, where $T_2^\star$ is the inhomogeneous dephasing time which is related to the root-mean-squared charge noise by

$$\sigma_\epsilon = \frac{1}{\sqrt{2}\pi T_2^\star} \cdot |\mathrm{d}f/\mathrm{d}\epsilon|^{-1}. \tag{31}$$

$T_2^\star$ is found for each detuning point by fitting the oscillations to Gaussian decay as shown in Fig. 4(a) of the main text. A functional form of $f(\epsilon)$ is found by fitting the data in Fig. 4(c) to a smooth function. We approximate the exchange energy as $J(\epsilon) \approx t_c^2/4\epsilon$, where t_c is the full-gap, inter-dot tunnel coupling, and find a good fit to $f = \frac{1}{h}\sqrt{J(\epsilon)^2 + \Delta_{\mathrm{SO}}^2}$. We extract $t_c = 0.7$ μeV from the fit. From the ratio of $T_2^\star(\epsilon)$ to $|\mathrm{d}f/\mathrm{d}\epsilon|^{-1}$, for detunings less than 30 μeV, a charge noise figure of $\sigma_e = 2.0 \pm 0.6$ μeV can be extracted. This value agrees with reported charge noise numbers of a few μeV[18, 24, 26, 27], indicating that proximity to the MOS interface does not degrade the qubit.

$T_{2\mathrm{m}}^*$ *Magnitude*

Several theoretical estimates of $T_2^\star$ in isotopically enriched silicon have been presented in the literature[28–30]. The estimate by Assali et. al. gives a $T_2^\star$ of 4.4 μs for the corresponding isotopic enrichment used in our experiments (500 ppm). Note: We have included a factor of 2 because the calculations in Ref. [28] do not account for $I = \frac{1}{2}$ of the ^{29}Si nuclei. Witzel et. al., on the other hand, predict a $T_2^\star$ of a few tens of μs, though they use a substantially larger QD radius. Following the central limit theorem, we expect $T_2^\star \sim \sqrt{N_S}$, where $\sqrt{N_S}$ is the number of spinful nuclei within the QD wavefunction, and that a decrease in QD size will lead to a decrease in the inhomogeneous dephasing time.

Furthermore, these reports consider single quantum dots. We are concerned with a DQD, in which each QD has a separate distribution of nuclear spins and the changes in the difference in hyperfine fields between QDs leads to the ST dephasing. Therefore, $T_2^\star$ is inversely proportional to the amount of fluctuations in the surrounding hyperfine field. If we say that each quantum dot has a normal distribution of hyperfine fields of the form

$$P = \frac{e^{-(x-\mu)^2/(\sigma_{\mathrm{QD}}^2)}}{\sigma_{\mathrm{QD}}^2\sqrt{2\pi}}, \tag{32}$$

where σ_{QD} is the variance in hyperfine field and μ is the average hyperfine field, then the distribution of the difference in hyperfine field between the two dots is given by,

$$P_{\mathrm{QD}_1-\mathrm{QD}_2} = \int_{-\infty}^{\infty}\int_{-\infty}^{\infty} \frac{e^{-x^2/(\sigma_{\mathrm{QD}_1}^2)}}{\sigma_{\mathrm{QD}_1}^2\sqrt{2\pi}} \frac{e^{-x^2/(\sigma_{\mathrm{QD}_2}^2)}}{\sigma_{\mathrm{QD}_2}^2\sqrt{2\pi}} \delta((x-y)-u)dxdy = \frac{e^{-|u-(\mu_{\mathrm{QD}_1}-\mu_{\mathrm{QD}_2})^2/[(\sigma_{\mathrm{QD}_1}^2)+\sigma_{\mathrm{QD}_2}^2]}}{\sqrt{2\pi(\sigma_{\mathrm{QD}_1}^2)+\sigma_{\mathrm{QD}_2}^2)}}. \tag{33}$$

This is a normal distribution with a variance of $\sqrt{\sigma_{\mathrm{QD}_1}^2 + \sigma_{\mathrm{QD}_2}^2}$ and an average difference in Hyperfine field of $(\mu_{\mathrm{QD}_1} - \mu_{\mathrm{QD}_2})$. Thus, we expect

$$T_{2,\mathrm{DQD}}^* = T_{2,\mathrm{QD}}^*/\sqrt{2}, \tag{34}$$

assuming similar sized QDs. Taking into account the differences in QD size and the effect of two QDs, which both imply a reduction in $T_2^\star$, our measured value of 1.6 μs fits well with these order of magnitude estimates.

Measurement Time Dependence of $T_{2\mathrm{m}}^*$

Figure 3(b) of the main text displays a clear dependence of $T_2^\star$ on the total experimental measurement time. This effect has been reported previously[24, 25], and is due to the time dynamics of the random hyperfine field from the residual 500 ppm ^{29}Si in the isotopically purified silicon host. Fluctuations in the polarization of the nuclei lead to varying magnetic fields at each QD between each experiment cycle. This leads to a varying qubit rotation frequency in each experimental cycle, which, as they are averaged together, lead to a decay in oscillation amplitude. As the experiment is measured for longer times, a larger sample of random nuclei polarization configurations, and a correspondingly larger distribution of qubit rotation frequencies is sampled.

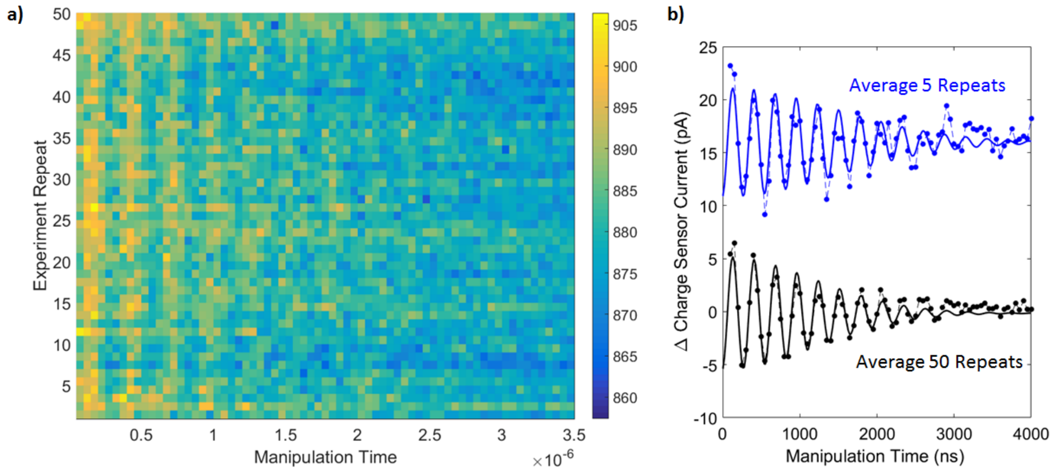

Supplementary Figure 5. Measurement Time Dependence. a) A repeated scan of charge sensor current vs. manipulation time for a magnetic field of 0.20 T along the [1$\bar{1}$0] crystallographic direction. b) Singlet-triplet rotation decay plots. In black, all 50 scans are averaged and a fit to the data gives a T_2^* of 1.66 µs. When only 5 line scans are averaged (blue curve, shifted by 17 pA for clarity) a T_2^* of 2.09 µs is extracted.

To obtain the plot in Figure 3(b), we repeat a measurement of charge sensor current versus manipulation time many times. An example of such a plot is shown in Supplementary Figure 5(a) for a magnetic field of 0.2 T along the [110] direction. By averaging various numbers of line scans together, we can examine the effect of measurement time on $T_2^\star$. Here, the total measurement time is the time for one experimental line scan times the number of scans averaged, and $T_2^\star$ is extracted by fitting the envelope of the averaged data to a Gaussian decay ($\exp[-(t/T_2^\star)^2]$). Examples of the averaged data for averaging 5 and 50 line scans are shown in Supplementary Figure 5(b).

Hahn-Echo Measurements

The experiments presented in this work indicate that the dephasing of the qubit during rotations is predominantly due to low-frequency, quasi-static noise. Dynamical decoupling techniques may be used to prolong qubit coherence. This method effectively filters the noise, such that qubit dephasing is most sensitive to noise around the experimental manipulation time. In this work we use a Hahn echo technique [31] to decouple from low frequency charge and magnetic noise. To perform a charge noise Hahn echo, a pulse sequence as detailed in Ref. [24] and depicted in Supplementary Figure 6(a) is used. For the results presented in the main text, we operated the qubit at a magnetic field of 0.141 T along the [110] direction giving an X-rotation frequency of $\Delta_{\mathrm{SO}}/h = 2.03$ MHz. We investigate the effect of a Hahn echo pulse sequence on charge noise decoherence at a detuning where the qubit rotation frequency is 2.24 MHz, corresponding to $J/h = 0.99$ MHz ($f = \sqrt{J^2 + \Delta_{\mathrm{SO}}^2}$). In Supplementary Figure 6(b) the measured echo signal is plotted as a function of the difference in evolution times for the first and second J-pulse (τ' - τ) for several total evolution times ($\tau' + \tau$). Here we have subtracted the background charge sensor current, leaving the echoed signal. The echo displays oscillations at 2.24 MHz and an overall Gaussian envelope corresponding to the inhomogeneous dephasing time. $T_2^\star$. By fitting the envelope to the form $A\exp[-((\tau' - \tau')/T_2^\star)^2]$, we can extract the echo amplitude, A, and the dephasing time, $T_2^\star$. We find an average dephasing time $T_2^\star = 1.02 \pm 0.06$ µs. In Supplementary Figure 6(b) we plot the extracted echo amplitude as a function of the total evolution times ($\tau + \tau'$). The data reveals a clear decay in amplitude with a characteristic $1/e$ decoherence time of $T_{2\mathrm{e}}^{\mathrm{echo}} \sim 8.4$ µs. This is comparable to results observed in GaAs/AlGaAs[24] and Si/SiGe[25] ST qubits.

Similar pulse sequences may be used to decouple the qubit from low-frequency magnetic noise. As shown in Supplementary Figure 7(a), we use a π pulse about the combined J and Δ_{SO} axis to create a Hahn-echo. In Supplementary Figure 7(b) the measured echo signal is plotted as a function of total evolution time under Δ_{SO}, $\tau' + \tau$, for a B = 0.2 T along the [100] crystallographic direction ($\Delta_{\mathrm{SO}}/h \sim 0.5$ MHz). An exponential fit reveals a $1/e$ decay time of $T_{2\mathrm{m}}^{\mathrm{echo}} \sim 70$ µs. The measured $T_{2\mathrm{m}}^{\mathrm{echo}}$ for several magnetic field strengths along the [100] is plotted in Supplementary Figure 7(c). This value is shorter than other times reported for $T_{2\mathrm{m}}^{\mathrm{echo}}$ in silicon[25]. T_2^{echo} may be bounded by excitation to higher energy states or other T_1 processes, and further experiments are required to reveal the limiting mechanism. However, this result illustrates our ability to extend coherence times through dynamical

decoupling and demonstrates our full two-axis control over the qubit.

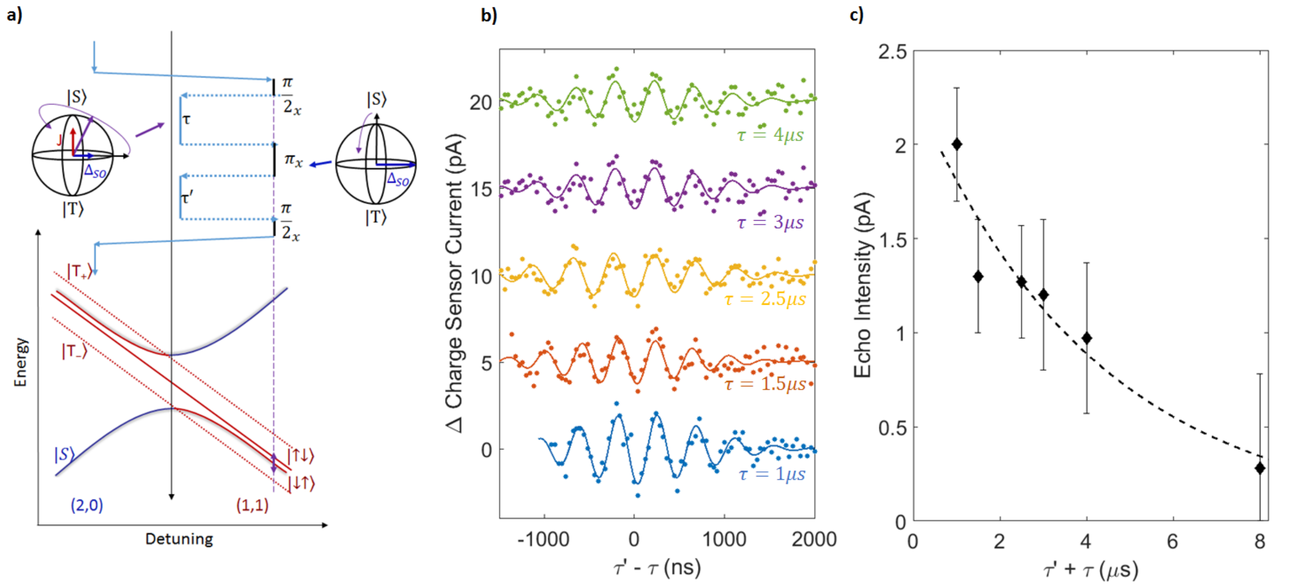

Supplementary Figure 6. Charge Noise Hahn Echo. a) The qubit is initialized in the S(2,0) ground state and a rapid adiabatic pulse transfers the system to the (1,1) charge sector such that it remains in a singlet state. The state is allowed to evolve for some time corresponding to $\pi/2$ pulse about the X-axis and rotates the spin state to the equator of the Bloch sphere. A pulse to a detuning, ϵ, where J is substantial for some time τ which causes the qubit to rotate about an axis depending on both J and $\Delta_{\rm SO}$ at a frequency $f = \sqrt{(J(\epsilon))^2 + \Delta_{\rm SO}^2}$. Here the qubit is susceptible to charge noise and, as a consequence, begins to dephase. A π pulse about the X-axis flips the spin across the Bloch sphere where, upon returning to detuning ϵ, the dephased qubit states refocus for a time τ'. A final $\pi/2$-pulse around the X-axis returns the qubit to the ST basis and a rapid adiabatic return pulse projects the states onto the $S(2,0)$ and $T_0(1,1)$ basis for measurement. b) A Hahn-echo return for several τ values along with fits to a Gaussian envelope function. c) Hahn-echo amplitude as a functions of total time evolving under the effect of charge noise $(\tau' + \tau)$. The error bars represent 95% confidence interval. The dashed line is a fit to an exponential decay.

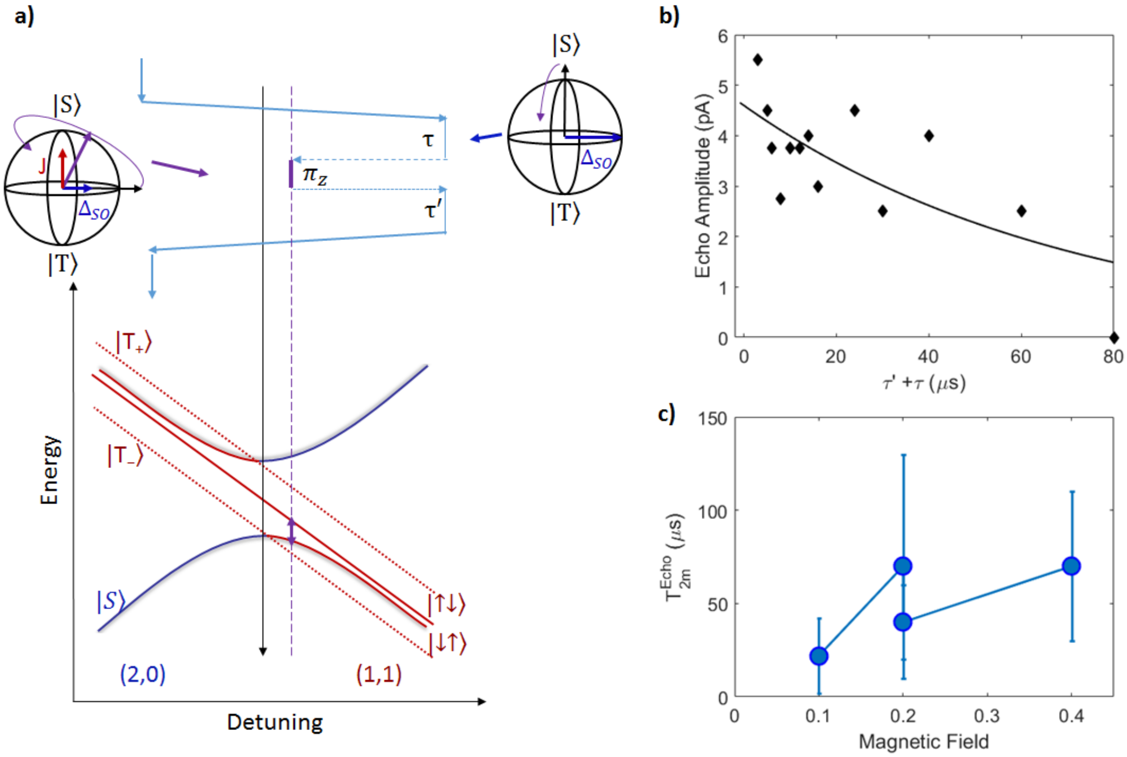

Supplementary Figure 7. Magnetic Noise Hahn-Echo. a) The qubit is initialized in the S(2,0) ground state and a rapid adiabatic pulse transfers the system to the (1,1) charge sector such that it remains in a singlet state where the state is allowed to evolve for some time, τ, about the X-axis under the influence of noise from magnetic fluctuations. A pulse to and from a detuning, ϵ, where J is substantial for a time corresponding to a π rotation about the axis depending on both J and $\Delta_{\rm SO}$ flips the spin across the Bloch sphere. The qubit states then evolve again for a time τ, refocusing the dephased qubit states. A rapid adiabatic return pulse projects the states onto the $S(2,0)$ and $T_0(1,1)$ basis for measurement. b) Hahn-echo amplitude as a functions of total time $(\tau + \tau)$ at a magnetic field of 0.2 T along the [100] crystallographic direction. The line is a fit to an exponential decay. c) The extracted $T_{\rm 2m}^{echo}$ for several magnetic field values along the [100] direction. The error bars represent 95% confidence interval.

SUPPLEMENTARY REFERENCES.

[1] Kawakami, E., Scarlino, P., Ward, D. R., Braakman, F. R., Savage, D. E., Lagally, M. G., Friesen, M., Coppersmith, S. N., Eriksson, M. A. & Vandersypen, L. M. K. Electrical control of a long-lived spin qubit in a Si/SiGe quantum dot. *Nat. Nano* **9**, 666–670 (2014).

[2] Veldhorst, M., Hwang, J. C. C., Yang, C. H., Leenstra, A. W., de Ronde, B., Dehollain, J. P., Muhonen, J. T., Hudson, F., Itoh, K. M., Morello, A. & Dzurak, A. S. An addressable quantum dot qubit with fault-tolerant control-fidelity. *Nat. Nano* **9**, 981–985 (2014).

[3] Veldhorst, M., Ruskov, R., Yang, C. H., Hwang, J. C. C., Hudson, F. E., Flatté, M. E., Tahan, C., Itoh, K. M., Morello, A. & Dzurak, A. S. Spin-orbit coupling and operation of multivalley spin qubits. *Phys. Rev. B* **92**, 201401 (2015).

[4] Ferdous, R., Kawakami, E., Scarlino, P., Nowak, M. P., Ward, D. R., Savage, D. E., Lagally, M. G., Coppersmith, S. N., Friesen, M., Eriksson, M. A., Vandersypen, L. M. K. & Rahman, R. Valley dependent anisotropic spin splitting in silicon quantum dots Preprint at http://arxiv.org/abs/1702.06210 (2017).

[5] Ferdous, R., Chan, K. W., Veldhorst, M., Hwang, J., Yang, C. H., Klimeck, G., Morello, A., Dzurak, A. S. & Rahman, R. Interface induced spin-orbit interaction in silicon quantum dots and prospects of scalability Preprint at http://arxiv.org/abs/1703.03840 (2017).

[6] Ruskov, R., Veldhorst, M., Dzurak, A. S. & Tahan, C. Electron g-factor of valley states in realistic silicon quantum dots Preprint at http://arxiv.org/abs/1708.04555 (2017).

[7] Nestoklon, M. O., Golub, L. E. & Ivchenko, E. L. Spin and valley-orbit splittings in SiGeSi heterostructures. *Phys. Rev. B* **73**, 235334 (2006).

[8] Nestoklon, M. O., Ivchenko, E. L., Jancu, J.-M. & Voisin, P. Electric field effect on electron spin splitting in SiGeSi quantum wells. *Phys. Rev. B* **77**, 155328 (2008).

[9] Prada, M., Klimeck, G. & Joynt, R. Spin–orbit splittings in si/sige quantum wells: from ideal si membranes to realistic heterostructures. *New Journal of Physics* **13**, 013009 (2011).

[10] Gamble, J. K., Harvey-Collard, P., Jacobson, N. T., Baczewski, A. D., Nielsen, E., Maurer, L., Montaño, I., Rudolph, M., Carroll, M. S., Yang, C. H., Rossi, A., Dzurak, A. S. & Muller, R. P. Valley splitting of single-electron si mos quantum dots. *Applied Physics Letters* **109**, 253101 (2016).

[11] Golub, L. E. & Ivchenko, E. L. Spin splitting in symmetrical sige quantum wells. *Phys. Rev. B* **69**, 115333 (2004).

[12] Gamble, J. K., Jacobson, N. T., Nielsen, E., Baczewski, A. D., Moussa, J. E., Montaño, I. & Muller, R. P. Multivalley effective mass theory simulation of donors in silicon. *Physical Review B* **91** (2015).

[13] Friesen, M., Chutia, S., Tahan, C. & Coppersmith, S. N. Valley splitting theory of SiGeSiSiGe quantum wells. *Phys. Rev. B* **75**, 115318 (2007).

[14] Stepanenko, D., Rudner, M., Halperin, B. I. & Loss, D. Singlet-triplet splitting in double quantum dots due to spin-orbit and hyperfine interactions. *Phys. Rev. B* **85**, 075416 (2012).

[15] Rochette, S., Rudolph, M., Roy, A.-M., Curry, M., Ten Eyck, G., Manginell, R., Wendt, J., Pluym, T., Carr, S. M., Ward, D., Lilly, M. P., Carroll, M. S. & Pioro-Ladrière, M. Single-electron-occupation metal-oxide-semiconductor quantum dots formed from efficient poly-silicon gate layout Preprint at http://arxiv.org/abs/1707.03895 (2017).

[16] Johnson, A. C., Petta, J. R., Marcus, C. M., Hanson, M. P. & Gossard, A. C. Singlet-triplet spin blockade and charge sensing in a few-electron double quantum dot. *Phys. Rev. B* **72**, 165308 (2005).

[17] Harvey-Collard, P., D'Anjou, B. T., Rudolph, M., Jacobson, N., Dominguez, J., Ten Eyck, G. A., Wendt, J. R., Pluym, T., Lilly, W. A., Michael Pand Coish, Pioro-Ladrière, M. & Carroll, M. S. High-fidelity single-shot readout for a spin qubit via an enhanced latching mechanism Preprint at http://arxiv.org/abs/1703.02651 (2017).

[18] Petersson, K., J.R., P., H, L. & A.C., G. Quantum coherence in a one-electron semiconductor charge qubit. *Physical Review Letters* **105**, 246804 (2010).

[19] Studenikin, S. A., Thorgrimson, J., Aers, G. C., Kam, A., Zawadzki, P., Wasilewski, Z. R., Bogan, A. & Sachrajda, A. S. Enhanced charge detection of spin qubit readout via an intermediate state. *Applied Physics Letters* **101**, 233101 (2012).

[20] Mason, J. D., Studenikin, S. A., Kam, A., Wasilewski, Z. R., Sachrajda, A. S. & Kycia, J. B. Role of metastable charge states in a quantum-dot spin-qubit readout. *Phys. Rev. B* **92**, 125434 (2015).

[21] Nakajima, T., Delbecq, M. R., Otsuka, T., Stano, P., Amaha, S., Yoneda, J., Noiri, A., Kawasaki, K., Takeda, K., Allison, G., Ludwig, A., Wieck, A. D., Loss, D. & Tarucha, S. Robust single-shot spin measurement with 99.5dot array. *Phys. Rev. Lett.* **119**, 017701 (2017).

[22] Broome, M. A., Watson, T. F., Keith, D., Gorman, S. K., House, M. G., Keizer, J. G., Hile, S. J., Baker, W. & Simmons, M. Y. High-fidelity single-shot singlet-triplet readout of precision-placed donors in silicon. *Phys. Rev. Lett.* **119**, 046802 (2017).

[23] Harvey-Collard, P., Jacobson, N. T., lph, M., Dominguez, J., Ten Eyck, G. A., Wendt, J. R., Pluym, T., Gamble, J. K., Lilly, M. P., Pioro-Ladrière, M. & Carroll, M. S. Coherent coupling between a quantum dot and a donor in silicon. *Nat. Commun.* **8**, 1029 (2017).

[24] Dial, O. E., Shulman, M. D., Harvey, S. P., Bluhm, H., Umansky, V. & Yacoby, A. Charge Noise Spectroscopy Using Coherent Exchange Oscillations in a Singlet-Triplet Qubit. *Physical Review Letters* **110**, 146804 (2013).

[25] Eng, K., Ladd, T. D., Smith, A., Borselli, M. G., Kiselev, A. A., Fong, B. H., Holabird, K. S., Hazard, T. M., Huang, B., Deelman, P. W., Milosavljevic, I., Schmitz, A. E., Ross, R. S., Gyure, M. F. & Hunter, A. T. Isotopically enhanced triple-quantum-dot qubit. *Sci. Adv.* **1**, e1500214 (2015).

[26] Shi, Z., Simmons, C. B., Ward, D. R., Prance, J. R., Mohr, R. T., Koh, T. S., Gamble, J. K., Wu, X., Savage, D. E., Lagally, M. G., Friesen, M., Coppersmith, S. N. & Eriksson, M. A. Coherent quantum oscillations and echo measurements of a si charge qubit. *Phys. Rev. B* **88**, 075416 (2013).

[27] Wu, X., Ward, D. R., Prance, J. R., Kim, D., Gamble, J. K., Mohr, R. T., Shi, Z., Savage, D. E., Lagally, M. G., Friesen, M., Coppersmith, S. N. & Eriksson, M. A. Two-axis control of a singlet–triplet qubit with an integrated micromagnet. *Proceedings of the National Academy of Sciences* **111**, 11938–11942 (2014).

[28] Assali, L. V. C., Petrilli, H. M., Capaz, R. B., Koiller, B., Hu, X. & Das Sarma, S. Hyperfine interactions in silicon quantum dots. *Phys. Rev. B* **83**, 165301 (2011).

[29] Witzel, W. M., Rahman, R. & Carroll, M. S. Nuclear spin induced decoherence of a quantum dot in si confined at a sige interface: Decoherence dependence on ^{73}Ge. *Phys. Rev. B* **85**, 205312 (2012).

[30] Witzel, W. M., Carroll, M. S., Cywiński, L. & Das Sarma, S. Quantum decoherence of the central spin in a sparse system of dipolar coupled spins. *Phys. Rev. B* **86**, 035452 (2012).

[31] Hahn, E. L. Spin echoes. *Phys. Rev.* **80**, 580–594 (1950).
